# Plasma Metabolite Profiles of Exercising American Foxhound Dogs Fed Different Diets

**DOI:** 10.3390/metabo16060397

**Published:** 2026-06-08

**Authors:** Sara E. Martini, Maria R. C. de Godoy, Alison N. Beloshapka, Preston R. Buff, Kelly S. Swanson

**Affiliations:** 1 Department of Animal Sciences, University of Illinois Urbana-Champaign, Urbana, IL 61801, USA; 2 The Nutro Company, Franklin, TN 37067, USA; 3 Division of Nutritional Sciences, University of Illinois Urbana-Champaign, Urbana, IL 61801, USA; 4 Department of Veterinary Clinical Medicine, University of Illinois Urbana-Champaign, Urbana, IL 61801, USA

**Keywords:** canine nutrition, canine metabolome, oxidative stress, exercise performance, plasma metabolomics

## Abstract

**Background/Objectives:** Canine athletes have a higher energy requirement and are more susceptible to nutrient depletion, electrolyte imbalance, and metabolic stress than sedentary pets. The objective of this study was to characterize the plasma metabolome of American Foxhound dogs following a bout of unstructured exercise. **Methods:** Thirty-nine adult American Foxhound dogs (32 intact males, 7 spayed females; age: 6.2 ± 3.1 yr; BW: 36.3 ± 5.3 kg) were allotted to a standard performance diet (CTRL) or NUTRO^®^ Natural Choice^®^ Adult High Endurance Formula (TEST). After 80 d in the study, blood samples were collected prior to (0 h), and 3 h and 25 h post-exercise (average: 17.7 km run over 2–3 h). Plasma samples of the 10 top performers of each treatment group were analyzed for untargeted metabolite profiling. **Results:** Of the 566 named metabolites identified, >200 and >185 metabolites were impacted (*p* < 0.05) by exercise and diet, respectively. Principal component analysis indicated distinct clustering by diet. Random forest analysis highlighted several metabolites having a high degree of predictive accuracy based on diet and exercise, with most related to amino acid, lipid, xenobiotic, and cofactor and vitamin metabolism. Relating to exercise, glycolytic end-products and citric acid cycle intermediates were increased at 3 h post-exercise. Similarly, tocopherols and omega-3 polyunsaturated fatty acids were higher in dogs fed TEST than those fed CTRL during recovery, indicating a lower oxidative stress and anti-inflammatory response. **Conclusions:** Overall, the data suggest a protective effect (lower susceptibility to oxidative stress and muscle fatigue) of feeding a nutrient-fortified diet for dogs undergoing unstructured exercise.

## 1. Introduction

To support exercise and maintain performance, athletic dogs have greater nutrient requirements that can be influenced by breed, body weight, and exercise duration compared to sedentary dogs [1,2]. Working dogs repeatedly perform bouts of endurance-based exercise for a variety of tasks (i.e., racing, hunting, herding, police/patrol); however, prolonged exercise may increase rates of oxidative stress and muscle damage, while depleting nutrient stores at the expense of sustaining activity.

Defined as prolonged periods of exercise, endurance exercise relies on muscular and cardiovascular stamina, as well as nutrient stores or production of intermediates through aerobic or anaerobic metabolism. Provided the lengthy time periods, endurance relies on mostly aerobic metabolism and lipids as the predominant energy source compared to short-term high-intensity exercise, which relies on anaerobic metabolism using carbohydrates as a primary substrate to produce the required ATP for muscle function [3]. In general, exercise notably increases the rate of skeletal and heart muscle contractions as well as the amount of oxygen consumed, thereby increasing the production of reactive oxygen species (ROS; free radicals) during oxygen metabolism, specifically at a subcellular level in muscular tissue [4]. Antioxidant mechanisms can assist with lowering concentrations of ROS but when these mechanisms are overwhelmed, oxidative stress occurs and can result in secondary radical species production during lipid peroxidation [4] as well as exercise-induced or long-term muscle damage with potential alterations in performance [5]. Therefore, dietary supplementation of antioxidants may have a protective effect against oxidative stress as summarized by Powers and Jackson [4] and have briefly been studied in exercising sled dogs [6,7]; however, further research is needed to confirm ideal antioxidant inclusion to negate excessive oxidative stress consequences.

Research is limited in the area of exercising dogs, especially when it comes to systemic measures of nutrient metabolism. The use of untargeted metabolomics has markedly increased across nutrition- and sport-based research, as it allows for a comprehensive view of metabolites relating to lipid, carbohydrate, and amino acid (AA) metabolism in order to form less biased conclusions regarding the effects of dietary or exercise-based treatments [8,9]. Using a metabolomics approach may allow for the identification of potential targets or predictive markers for oxidative stress or metabolites to determine effects of short-term exercise on metabolism. Therefore, the objective of this study was to characterize the plasma metabolome of American Foxhound dogs following a bout of unstructured exercise. Based on previous research [10,11] we hypothesized that there would be lower concentrations of oxidative stress markers, with increased antioxidant concentrations and metabolites relating to AA metabolism in dogs fed a nutrient-fortified diet (i.e., higher dietary protein and AA, and increased antioxidant inclusion).

## 2. Materials and Methods

All animal procedures were approved by the Waltham Centre for Pet Nutrition Animal Ethics and Welfare Committee and verbal informed consent was obtained from the owner of the kennel (Hard Away Hounds) prior to experimentation.

### 2.1. Animals, Diets, and Experimental Design

Thirty-nine adult American Foxhound dogs [32 intact males, 7 spayed females; mean age = 6.2 ± 3.1 yr; mean BW = 36.3 ± 5.3 kg] as part of a previously conducted study [10]. Dogs were group-housed throughout the trial and separated by dietary treatment and sex.

All dogs were allowed 8 h in a play yard during the day and were provided at least 30 min of human interaction as social enrichment as a group. Outdoor runs contained trees for shade, or covered, raised platforms. Overnight and during inclement weather, all dogs were housed in a lodge, which provided outdoor access if the dogs preferred it, with suspended platforms and beds for animal use. The indoor facility had both natural and artificial lighting and was equipped with fans. Artificial lighting was used to maintain a light:dark cycle of 12:12 h and allowed morning feeding (05:00 h) and animal handling until later in the evening (17:00 h). All play yard and lodging spaces were cleaned daily.

Dietary treatments included a standard performance diet (Sportmix High Energy; Midwestern Pet Foods, Inc., Evansville, IN; control; *n* = 19) or NUTRO^®^ Natural Choice^®^ Adult High Endurance Formula (test; n = 20), which was formulated with higher inclusions of zinc, vitamins E and C, lutein, and taurine. The control diet provided 65.7 g protein and 51.0 g fat per 4184 kJ metabolizable energy (1 kcal = 4.184 kJ), respectively, and had a metabolizable energy of 17.9 kJ/g, based on the National Research Council equation [12]. The test diet provided greater protein (73.6 g) and fat (52.9 g) per 4184 kJ metabolizable energy and had a similar calculated metabolizable energy of 18.7 kJ/g [12]. A complete list of ingredients and guaranteed analysis of diets is presented in Supplementary Table S1.

### 2.2. Exercise Regimen and GPS Measurements

Dietary treatment assignments were balanced by sex, age, body weight and athletic performance. Dogs were fed their respective treatments for 80 d. Then, dogs were allowed to exercise for 2–3 h of unstructured mixed exercise that simulated chasing in a field or a herding field exercise. Movement was tracked via GPS collars (DC 40, Garmin Ltd., Olathe, KS, USA) and activity data were collected using Astro 320 Global Positioning System (Garmin Ltd.) to calculate total miles run and speeds per dog.

### 2.3. Blood Collection and Plasma Analysis

Venous blood samples were collected via jugular puncture prior to exercise (0 h) and 3 h and 25 h post-exercise into sodium heparin vacutainer blood tubes (Becton Dickson, Franklin Lakes, NJ, USA) and immediately placed on ice for approximately 30 min. Tubes were centrifuged at 1240× *g* for 10 min at 4 °C and then plasma samples were stored at −80 °C until analyses. Plasma samples from the top 10 performers (determined by distance run; control diet range: 16.3–26.9 km; and test diet range: 18.1–30.3 km) were selected for this analysis, as this was unstructured exercise voluntarily performed by the dogs. To more effectively quantify the effects of exercise, the top 10 performers from each dietary allotment were selected because these dogs had greater duration of exercise compared with the entire test population. The top performing plasma samples were sent to Metabolon (Metabolon, Inc., Durham, NC, USA) for untargeted metabolite profiling.

Using similar methods to Pallotto et al. [13], samples were shipped on dry ice to Metabolon and immediately stored at −80 °C upon arrival. Each sample was inventoried into the Metabolon Laboratory Information Management System (LIMS) and assigned a unique identifier, which was used to track all sample handling, tasks, and results. All samples were maintained at −80 °C until processed. Samples were prepared using the automated MicroLab Star^®^ system from Hamilton Company (Salt Lake City, UT, USA). A recovery standard was added prior to the first step in the extraction process for quality control purposes. To remove protein, small molecules bound to protein or trapped in the precipitated protein matrix were dissociated, and to recover chemically diverse metabolites, proteins were precipitated with methanol under vigorous shaking for 2 min using a GenoGrinder 2000 (Glen Mills, NJ, USA) followed by centrifugation. The resulting extract was divided into four fractions: one for analysis by ultrahigh performance liquid chromatography-tandem mass spectrometry (UPLC-MS/MS) with positive ion mode electrospray ionization, one for analysis by UPLC-MS/MS with negative ion mode electrospray ionization, one for analysis by gas chromatography-mass spectrometry (GC-MS), and one sample reserved for backup. Samples were placed briefly on a TurboVap^®^ (Thermo Fisher Scientific Inc., Waltham, MA, USA) to remove the organic solvent. For LC, the samples were stored overnight under nitrogen before preparation for analysis. For GC, each sample was dried under vacuum overnight before preparation for analysis.

Three types of controls were analyzed in concert with the experimental samples: a pooled matrix sample generated by taking a small volume of each experimental sample (or alternatively, use of a pool of well-characterized human plasma) served as a technical replicate throughout the data set; extracted water samples served as process blanks; and a cocktail of quality control standards that were carefully chosen not to interfere with the measurement of endogenous metabolites were spiked into every analyzed sample, allowing for instrument performance monitoring and aiding chromatographic alignment. Instrument variability was determined by calculating the median relative standard deviation (RSD) for the standards that were added to each sample prior to injection into the mass spectrometers. The RSD for internal standards in this study was 5%. Overall process variability for this study was 14% and was determined by calculating the median RSD for all endogenous metabolites (i.e., non-instrument standards) present in 100% of the pooled matrix samples. Experimental samples were randomized across the platform run with quality control samples spaced evenly among the injections.

The LC/MS portion of the platform was based on a Waters ACQUITY ultra-performance liquid chromatography and a high-resolution/accurate mass spectrometer (Thermo Fisher Scientific) interfaced with a heated electrospray ionization (HESI-II) source and Orbitrap mass analyzer operated at 35,000 mass resolution. The sample extract was dried and then reconstituted in acidic or basic LC-compatible solvents, each of which contained 8 or more injection standards at fixed concentrations to ensure injection and chromatographic consistency. One aliquot was analyzed using acidic positive ion optimized conditions and the other using basic negative ion optimized conditions, in two independent injections using separate dedicated columns (Waters UPLC BEH C18-2.1 × 100 mm, 1.7 µm). Extracts reconstituted in acidic conditions were gradient eluted from a C18 column using water and methanol containing 0.1% formic acid. The basic extracts were similarly eluted from C18 using methanol and water, but with 6.5 mM ammonium bicarbonate. The third aliquot was analyzed via negative ionization, following elution from an HILIC column (Waters UPLC BEH Amide 2.1 × 150 mm, 1.7 µm) using a gradient consisting of water and acetonitrile with 10 mM ammonium formate. The MS analysis alternated between MS and data-dependent MS2 scans using dynamic exclusion, with the scan range of 80–1000 mass to charge ratio (*m*/*z*).

The samples used for analysis by GC-MS were dried under vacuum for a minimum of 18 h prior to being derivatized under dried nitrogen using bistrimethyl-silyltrifluoroacetamide. Derivatized samples were separated on a 5% diphenyl/95% dimethyl polysiloxane fused silica column (20 m × 0.18 mm ID; 0.18 μm film thickness) with helium as a carrier gas and a temperature ramp from 60 °C to 340 °C in a 17.5 min period. Samples were analyzed on a Thermo Finnigan Trace DSQ fast-scanning single-quadrupole mass spectrometer using electron impact ionization and operated at unit mass resolving power. The scan range was from 50–750 *m*/*z*.

Raw data were extracted, peak-identified, and QC processed using Metabolon’s hardware and software. These systems are built on a web-service platform utilizing Microsoft’s .NET technologies, which run on high-performance application servers and fiber-channel storage arrays in clusters to provide active failover and load-balancing. Metabolites were identified by comparison to library entries of purified standards or recurrent unknown entities. Metabolon maintains a library based on authenticated standards that contains the retention time/index (RI), *m*/*z*, and chromatographic data (including MS/MS spectral data) on all molecules present in the library. Furthermore, biochemical identifications are based on three criteria: retention index within a narrow RI window of the proposed identification, accurate mass match to the library +/− 0.005 amu, and the MS/MS forward and reverse scores between the experimental data and authentic standards. The MS/MS scores are based on a comparison of the ions present in the experimental spectrum to the ions present in the library spectrum. While there may be similarities between these molecules based on one of these factors, the use of all three data points can be utilized to distinguish and differentiate biochemicals. More than 3300 commercially available purified standard metabolites have been acquired and registered into LIMS for distribution to both the LC-MS and GC-MS platforms for determination of their analytical characteristics. Additional mass spectral entries have been created for structurally unnamed biochemicals, which have been identified by virtue of their recurrent nature (both chromatographic and mass spectral).

A variety of curation procedures were carried out to ensure that a high-quality data set was made available for statistical analysis and data interpretation. The quality control and curation processes were designed to ensure accurate and consistent identification of true chemical entities, and to remove those representing system artifacts, mis-assignments, and background noise. Library matches for each metabolite were checked for each sample and corrected if necessary. Peaks were quantified using area-under-the-curve. For studies spanning multiple days, a data normalization step was performed to correct variation resulting from instrument inter-day tuning differences. Each metabolite was corrected in run-day blocks by registering the medians to equal one (1.00) and normalizing each data point proportionately (termed the “block correction”). For studies that did not require more than one day of analysis, no normalization is necessary, other than for purposes of data visualization.

### 2.4. Statistical Analysis

Statistical analysis was conducted by Metabolon using ArrayStudio on log transformed data. Principal component analysis was performed using all named metabolites to provide a simultaneous comparison of metabolic alterations associated with dietary treatment and time post-exercise. Hierarchical clustering was used to show large-scale differences in metabolic patterns. Random forest analysis (RFA) was performed to provide an estimate of how well individuals may be classified in the dataset. For a given decision tree, a random subset of data were selected to build a tree (“bootstrap sample”), and the remaining data, the “out-of-bag” variables, were passed through the tree to obtain a class prediction for each sample. After the process was repeated thousands of times, a forest was produced. The final classification of each sample was determined by computing the class prediction frequency for the “out-of-bag” variables over the whole forest; therefore, the “out-of-bag” error rate is a measure of prediction accuracy. In this study, the metabolic profiles of dietary treatments at three timepoints (0 h, 3 h and 25 h) were compared. To determine which variables (metabolites) made the largest contribution to the classification, the mean decrease accuracy (MDA) was determined by randomly permuting a variable, running the observed values through the trees, and then reassessing the prediction accuracy. If a variable was important to the classification, the prediction accuracy dropped after such a permutation. Thus, the RFA provided an importance rank ordering of metabolites. The top 30 metabolites were reported for each comparison.

A one-way analysis of variance (ANOVA) with repeated measures identified markers that were altered with dietary treatment and time post-exercise. An estimate of a false discovery rate (q-value) was calculated to take into account multiple comparisons. A combination of *p*- and q-value ≤ 0.05 was used to declare statistical significance. Statistical analyses were performed using the program “R” (http://cran.r-project.org/) and JMP (SAS Inst. Inc., Cary, NC, USA).

## 3. Results

**Figure 1:**
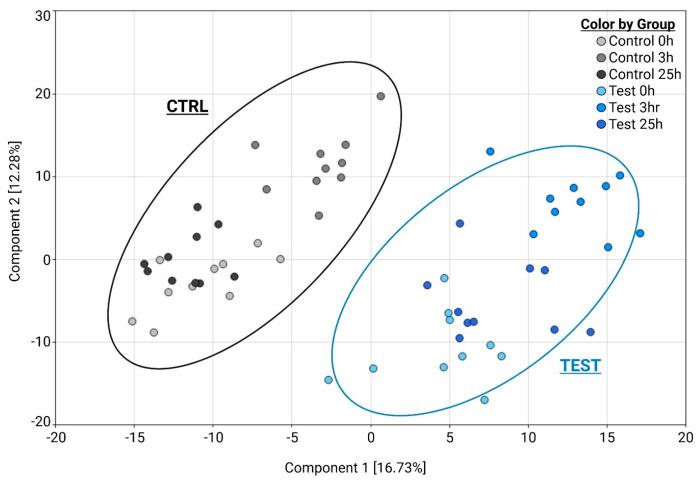
Principal component analysis of metabolite profiles of dogs consuming CTRL or TEST diet at all timepoints (0 h, 3 h, 25 h).

A total of 566 named metabolites were identified in this study. Principal component analysis comparing diet and time is presented in Figure 1, with distinct clustering within diet and less distinct effects relating to time post-exercise.

RFA was performed to identify and rank the top metabolites affected by diet (CTRL, TEST) and time prior to (0 h) and post-exercise (3 h, 25 h). Additionally, RFA demonstrated a high degree of predictive accuracy (97–100%) for all analyses. MDA was calculated to determine variable importance and the top 30 ranking metabolites key to distinct group separation are presented in Table 1 and Supplementary Figures S1–S5. To compare the effects of post-exercise status in CTRL-fed dogs (CTRL 0 h vs. 3 h vs. 25 h), RFA demonstrated the top differentiating metabolites with the top 30 presented in Table 1 and Supplementary Figure S1. Of these, 11/30 (36.7%) were involved in animo acid metabolism, followed by peptides (8/30; 26.%), xenobiotics (5/30; 16.7%), lipids (4/30; 13.3%) and cofactors and vitamins (2/30; 6.7%). The top 5 metabolites included dihydroferulic acid (xenobiotic), lipids (oleoyl-linoleoyl-glycerophosphocholine, oleate [18:1n9]), pterin (cofactor), and 2-hydroxybutyrate (peptide). Post-exercise, RFA analysis of dogs consuming TEST (TEST 0 h vs. 3 h vs. 25 h) differentiated the top plasma metabolites with predominant involvement in AA metabolism (15/30; 50%), xenobiotics (8/30; 26.7%), and peptide metabolism (5/30; 16.7%) (Table 1; Supplementary Figure S2).

To compare diet-related differences across exercise stages, RFA analysis was conducted comparing CTRL vs. TEST at 0 h (Supplementary Figure S3), 3 h (Supplementary Figure S4) and 25 h (Supplementary Figure S5), with the most significant metabolites being presented in Table 1. Initial baseline (0 h) differences between CTRL-fed and TEST-fed dogs demonstrated key metabolite differences related to lipid metabolism (9/30; 30%) and xenobiotics (7/30; 23.3%), followed by AA and cofactors and vitamins (5/30; 16.7% each) and peptide metabolism (4/30; 13.3%). Similar patterns were observed during analysis of CTRL vs. TEST at 3 h (Table 2), but with fewer relating to xenobiotic metabolism (4/30; 13.3%). However, 25 h post-exercise demonstrated greater distinction relating to AA metabolism (11/30; 36.7%) and peptide metabolism (6/30; 20%) and less distinction relating to lipid or xenobiotic metabolism.

Of the 566 named metabolites identified, over half of them (i.e., 386) were related to lipid or AA metabolism. Of the metabolites identified, more than 200 were significantly (*p* < 0.05) affected by diet and more than 350 were affected by exercise. Significant diet*time interactions were observed for more than 50 metabolites involved in metabolism of lysine, methionine/cysteine/SAM/taurine, creatine, polyunsaturated fatty acids (FAs), pantothenate and CoA, biotin, and vitamin B6 and are represented as fold change.

Plasma metabolites related to AA metabolism (branched-chain AA [BCAA], lysine, taurine, urea cycle via arginine and proline metabolism, and creatine) are presented in Table 3, many of which are also intermediates of the urea cycle. Of these, a significant diet*time interaction was observed for 22/68 identified metabolites. First, metabolites relating to BCAA metabolism showed mixed effects, with some higher in TEST than CTRL dogs (e.g., triglyl carnitine and N-acetylvaline at 3 h, methylsuccinate, isobutyrylcarnitine). Lysine was significantly higher in TEST-fed dogs, whereas N2- and N6-acetyllysine were lower across all time points (*p* < 0.05). At 3 h, 2-aminoadipate and glutarate (pentanedioate) were lower in CTRL-fed dogs (*p* < 0.05). Similar to lysine, homoarginine and creatine were significantly (*p* < 0.05) higher in TEST-fed dogs by 25 h, with arginine and urea tending (*p* < 0.10) to be higher after 25 h. Dimethylarginine, N-delta-acetylornithine, and N-methyl proline were all higher in CTRL-fed dogs at all time points. Finally, methionine, and taurine were higher (*p* < 0.05) in TEST-fed dogs compared with CTRL-fed dogs across all time points.

Time post-exercise contributed significantly to shifts in the metabolite profiles of dogs consuming either dietary treatment. Due to the abundance of metabolites identified, it is difficult to discuss individual effects; however, key patterns emerged when evaluating sub pathways. The first 3 h post-exercise demonstrated immediate and short-term (0 h vs. 3 h) differences via increases in metabolites relating to AA and nucleotide metabolism, whereas lipid and xenobiotic metabolism generally decreased as the majority of metabolites in those pathways had greater fold change reductions compared to 0 h post-exercise. Additionally, metabolites related to carbohydrate metabolism (e.g., glycolysis, gluconeogenesis and pyruvate metabolism) were also increased in the immediate short-term recovery (3 h), with increased glucose, lactate, pyruvate (lactate precursor), and glycerate (breakdown product of triglycerides), demonstrating the mobilization of glucose and energy reserves necessary to maintain blood glucose during muscle recovery. The longer-term (0 h vs. 25 h) recovery period was similar in pattern; however, there were fewer metabolites contributing to overall changes.

As exercise is known to increase oxidative stress markers, glutathione metabolism should be mentioned as a predominant outcome as it plays a vital role in limiting oxidative stress in the blood. Five metabolites were quantified in relation to glutathione metabolism including oxidized glutathione (GSSG), cysteine-glutathione disulfide, S-methylglutathione, 5-oxoproline, and opthalmate. Each were significantly affected by time and increased when comparing 0 h vs. 3 h regardless of dietary treatment (Table 3). Specifically comparing 3 h to 25 h, all glutathione metabolites were decreased at 25 h in TEST-fed and CTRL-fed dogs, except for GSSG in CTRL which was not affected. Lastly, opthalmate was decreased in TEST-fed dogs when compared to CTRL-fed dogs across all time points (*p* = 0.0054).

To demonstrate baseline dietary differences, ANOVA contrasts comparing TEST to CTRL at 0 h revealed 185 metabolites were significantly (*p* < 0.05) altered by dietary treatment, with a majority involved with lipid metabolism or AA metabolism. Metabolism of FAs (medium/long/branched chain, amino, acyl glycine, and monohydroxy) and bile acids (BA; primary, secondary) was greater in CTRL-fed dogs compared to TEST-fed dogs (Supplementary Table S2). Polyunsaturated FAs were greater in TEST-fed dogs whereas lysolipid metabolism greatly depended on the phospholipid structure. For example, lipids with two FA head groups (i.e., palmitic, stearic, or oleic acid) were lower in TEST-fed vs. CTRL-fed dogs, whereas lipids with only one FA were generally higher in TEST-fed dogs. Metabolites relating to AA metabolism were lower in TEST-fed dogs (34/51), with increased methionine, cysteine, taurine, isoleucine, and histidine metabolism compared to CTRL-fed dogs (Supplementary Table S3). Of the other super pathways, peptides and xenobiotics (benzoate and food-related) were significantly lower (*p* < 0.05) in TEST-fed dogs (Supplementary Table S4). Additionally, metabolites related to carbohydrate metabolism (pyruvate, fructose, mannitol, and glucoronate) were also lower in TEST-fed dogs at baseline. Lastly, all of the significant cofactors and vitamins were increased as a result of the TEST diet.

Relating to dietary influence, several metabolites were altered under the cofactor and vitamin super pathways and are presented in Table 4. Nicotinamide metabolites, pantothenate, several critical antioxidant tocopherols, biotin, and vitamin B6 (pyridoxal, pyridoxate) metabolites were significantly (*p* < 0.05) increased in TEST-fed compared to CTRL-fed dogs. More specifically and after 25 h post-exercise, sustained increases of these metabolites were also observed to suggest that TEST-fed dogs retained greater redox potential (via nicotinamides) and oxidative defenses (riboflavin, alpha- and gamma-tocopherol and catabolite precursors CEHC-glucuronide).

Additionally, several metabolites were altered due to diet under lipid metabolism, including many lysolipids, FAs (medium, long, and branched chain, amino, dicarboxylate, and monohydroxy), cholesterol, omega-3 FAs (eicosapentaenoate [EPA] and docosahexaenoate [DHA]), and primary and secondary BAs (Table 5, Supplementary Table S5). In general, EPA and DHA were consistently higher in TEST-fed dogs compared to CTRL-fed dogs, with reduced omega-6 FAs (i.e., linoleate and arachidonate), with mixed effects on lysolipids and other polyunsaturated FA. Opposite effects were observed with cholesterol and primary and secondary BA, which were lower in TEST-fed dogs in comparison to CTRL-fed dogs. Interestingly, some of the FA metabolites (e.g., dicarboxylate, monohydroxy) were found to be increased in CTRL-fed dogs regardless of exercise status, potentially serving as intermediates for mitochondrial metabolism at a basal level. Several xenobiotic metabolites were also altered with dietary treatment and are presented in Supplementary Table S6. In brief, the majority of identified benzoate metabolites (i.e., 3- and 4-methyl catechol sulfate, 4-ethylphenylsulfate) and many food component/plant metabolites, including equol sulfate and erythritol, were lower in TEST-fed dogs when compared to CTRL-fed dogs. Mixed effects were observed with drug- or chemical-related xenobiotics.

## 4. Discussion

The objective of the present study was to identify biomarkers related to oxidative stress and nutrient metabolism using the plasma collected from American Foxhounds subjected to unstructured mixed exercise to observe the metabolic effects of feeding a nutrient-fortified diet. Biomarkers were identified via metabolome analysis, which provided a snapshot view of metabolic processes to develop ideas on how nutrients are processed and where key metabolic areas are as pertaining to exercise. As part of this analysis, more than 500 metabolites were identified, with >200 affected by exercise and >185 metabolites altered due to diet. The main metabolites altered between diets and over time related to metabolic pathways of AA, peptides, xenobiotics, lipids, and cofactors and vitamins, many of which are discussed below.

Commonly observed with high-intensity exercise is the development of muscle soreness during recovery due to the muscular damage accumulated from rapid muscle contractions and metabolic reactions required to sustain activity. BCAAs (leucine, isoleucine, and valine) are catabolized in the skeletal tissue and predominantly contribute to muscle growth and repair post-exercise, as these are directly incorporated into skeletal muscle and have become increasingly popular for supplemental use to aid in human post-exercise recovery [14,15]. Therefore, BCAAs are important in muscle recovery via protein turnover and synthesis and were recently reviewed as a beneficial supplement for athletes [16]. BCAA supplementation is thought to limit the effects of creatine kinase, an indirect marker of muscle damage contributing to muscle soreness [17] and may accelerate recovery rates. In a previously conducted study [9], dogs fed the same test diet demonstrated a greater plasma taurine and branched-chain AA:tryptophan ratio post-exercise, which has previously been shown to delay mental and physical fatigue throughout intense exercise [18] in addition to delaying onset of muscle soreness when evaluated up to 96 h post-exercise in humans [19]. Of the BCAAs, leucine is primarily involved with protein synthesis [20] and was higher in TEST-fed dogs post-exercise, in addition to taurine.

In human-based sports research, there is inconclusive evidence as to whether or not taurine supplementation is effective during exercise performance, metabolic stress, muscle recovery or soreness [21]. However, adequate taurine status is important in maintaining cardiac function [22] and was higher in TEST-fed dogs in this study. It is important to note that taurine inclusion was higher in the test diet and may explain the observed increase. Large breed dogs are at risk of developing dilated cardiomyopathy [23] and so ensuring adequate and available taurine in the diet is vital to avoid deficiency or disease development/progression [24,25], especially in those that are undergoing exercise. Despite the treatment differences, all dogs used for this follow-up study were above the taurine thresholds as discussed in de Godoy et al. [10].

During exercise of greater intensity, metabolic patterns shifts towards anaerobic metabolism to utilize carbohydrates to maintain performance [3]; however, this can increase free radical production [6] and markers of oxidative stress (i.e., GSSG). To determine the extent of oxidative stress, quantifying the amount of reduced glutathione (GSH; free radical scavenger) compared to GSSG can be useful. GSH was not one of the metabolites identified in the present analysis so it is difficult to conclude that it can be used for predicting exercise-related metabolism. However, GSSG was increased 3 h and 25 h post-exercise, indicating elevated oxidative stress. To protect from damage, inclusion of antioxidants (i.e., tocopherols, vitamins C and E, and lutein) in performance-based diets may be useful to prevent excessive ROS accumulation. For example, vitamins C and E can directly eliminate ROS and reduce lipid peroxidation to prevent further damage to cell membranes [26]. Research on antioxidant use in exercising dogs is limited, but in 62 Alaskan sled dogs supplemented daily with antioxidants(400 units alpha-tocopherol acetate, 3 mg beta-carotene, and 20 mg lutein), plasma antioxidant concentrations increased alpha-tocopherol (up to 3 d post-exercise) and lutein (one day post-exercise) and reduced markers of DNA damage [7]. In another study using sled dogs, daily supplementation of antioxidants (457 mg of vitamin E, 706 mg of vitamin C and 5.1 mg of β-carotene) failed to attenuate the exercised-induced metabolic changes [6]. In the present study, the extent of oxidative stress was limited (using GSSG as a marker) so it is hypothesized that the exercise regimen was not intense enough to induce excessive stress and that both diets provided sufficient antioxidants to minimize ROS production.

Interestingly, several B-vitamin metabolites were increased in TEST-fed dogs compared to CTRL dogs (i.e., riboflavin, pantothenate, biotin, pyridoxal, and pyridoxate) with known roles in oxidation–reduction reactions and methylation of homocysteine to methionine for involvement in DNA synthesis and repair. An insufficient amount of these cofactors can lead to an accumulation of homocysteine, which can increase inflammation and oxidative stress [27,28]. Therefore, it appeared that dogs fed TEST were protected from such effects post-exercise.

In addition to an increase in ROS, exercise can modulate the cytokine and inflammatory response due to muscle damage and the necessity for repair [28]. Dietary components, including omega-3 FAs such as EPA (20:5n3), DHA (22:6n3), and alpha-linolenic acid (18:3n3), have anti-inflammatory and protective effects as widely discussed in the literature. Pertaining to exercise, these FAs can also attenuate muscle and cartilage damage relating to exercise. As recently highlighted in a systematic review, the consumption of omega-3 FAs improved a few oxidative stress markers (GSH and GSSG) and decreased proinflammatory cytokines in athletes [29]. Further research should be conducted in exercising dogs to determine more ideal ranges of omega-3 FA inclusion, if at all different from current National Research Council guidelines (0.044% linolenic and EPA/DHA recommendations). Lastly, the present results demonstrated a decrease to the n-6:n-3 ratio, which is considered to be beneficial as this would suggest a shift towards anti-inflammatory conditions attributable to post-exercise recovery.

It should be discussed that a few of the metabolites identified in the present study may have been altered due to metabolism by the host or gastrointestinal microbiota (i.e., microbial-derived uremic toxins such as p-cresol sulfate and trimethylamine N-oxide; secondary BA production) given that these samples were analyzed using the plasma of canines. It is well-established that the gastrointestinal microbial composition and activity can vary significantly depending on many factors such as diet consumption [30], antibiotic/drug use [31], and age/health status [32]. Dietary influence remains to be one of the most powerful influencers as anything undigested or that bypasses host enzymatic digestion is susceptible to microbial digestion and fermentation. As microbial-derived metabolites are produced in the distal gastrointestinal tract and can be reabsorbed in the distal colon to re-circulate in the bloodstream, it is difficult to determine if the statistical significance was observed due to diet- or exercise-related metabolic differences or altered microbial activity.

As this study was conducted in conjunction with a previously published report [10], similar study limitations apply and should be addressed. First, individual food intake was not controlled or recorded and therefore limits the interpretation of dietary-related effects on metabolic metabolites. Also, both diets had unique nutrient compositions, which can create a challenge in determining exact nutrient-related effects. As a suggestion for future trials, formulating test diets balanced in all nutrient profiles and only manipulating nutrient-fortification via antioxidant inclusions would be beneficial to addressing this limitation. Additionally, the exercise regimen was unstructured, meaning that the intensity was not controlled across individuals so all animals may not have been challenged similarly. However, the top 10 performers were selected for this analysis to better assess the influence of exercise on plasma metabolites for biomarker identification. For future studies, more controlled and structured exercise would be ideal for repeatability. However, formal structured exercise may not be sufficiently representative of natural hunting dog behaviors. Future studies addressing these limitations would likely provide additional insight into further dietary recommendations for hunting dogs, especially when following bouts of exercise.

## 5. Conclusions

In summary, the metabolites identified in the present study suggest that feeding a nutrient-fortified diet (i.e., increased antioxidant inclusion) may improve systemic protection from oxidative stress and limit muscle fatigue in dogs undergoing unstructured exercise. Consumption of the nutrient-fortified diet distinctly shifted plasma metabolic composition and increased BCAAs, tocopherol metabolism (CEHC metabolites) and omega-3 FAs (EPA and DHA) while reducing omega-6 FAs (arachidonic acid) prior to exercise in dogs consuming the nutrient-fortified diet. Following exercise at 3 h and 25 h, intermediates of the TCA cycle (i.e., citrate, alpha-ketoglutarate, succinylcarnitine) and glycolytic end products (i.e., lactate, pyruvate, glucose) were increased, demonstrating the importance of accessible energy sources during initial exercise recovery. Finally, microbial-derived metabolites (i.e., hippurate, equol sulfate, 4-ethylphenylsulfate, and secondary BAs) were lower in dogs consuming the nutrient-fortified diet during exercise recovery. For future studies evaluating effects of exercise on plasma metabolics, potential biomarkers may include oxidative stress-related metabolites (i.e., GSSG, GSH, and B vitamins), antioxidant-involved metabolites (i.e, tocopherols), and anti-inflammatory compounds (i.e., omega-3 FAs).

## Figures and Tables

**Table 1:** Top 30 plasma metabolites differing between CTRL- and TEST-fed dogs prior to (0 h) and post-exercise (3 h and 25 h), according to RFA analysis.

CTRL 0 h vs. 3 h and 25 h	TEST 0 h vs. 3 h and 25 h	Metabolite Super Pathway
2-hydroxybutyrate *	3-indoxyl sulfate	Amino Acid
3-indoxyl sulfate	glutarate (pentanedioate) *	
3-methyl-2-oxovalerate	ophthalmate	
alpha-ketobutyrate	3-(4-hydroxyphenyl)lactate	
glutarate (pentanedioate)	3-hydroxyisobutyrate	
isobutyrylglycine	3-methyl-2-oxovalerate	Peptide
isovalerylglycine	4-methyl-2-oxopentanoate	
methylsuccinate	asparagine	
N-acetylglycine	creatine	
ophthalmate	glutamine	
phenylpyruvate	leucine	Carbohydrate
aspartylleucine	lysine	
cis-Cyclo[L-ala-L-Pro]	phenol sulfate	
cyclo(L-phe-D-pro)	phenylalanine	Lipid
cyclo(L-phe-L-pro)	valine	
gamma-glutamyl-2-aminobutyrate	aspartylleucine	
gamma-glutamylphenylalanine	gamma-glutamyl-2-aminobutyrate	
phenylalanyltryptophan	gamma-glutamylglutamine	Nucleotide
valylleucine	gamma-glutamylphenylalanine	
myristate (14:0)	gamma-glutamyltryptophan	
oleate (18:1n9) *	ribonate	
oleoyl-linoleoyl-glycerophosphocholine (1) *	urate	Cofactors and Vitamins
oleoyl-linoleoyl-glycerophosphocholine (2)	2-aminophenol sulfate	
1-methylnicotinamide	2-hydroxyhippurate (salicylurate) *	
pterin *	2-piperidinone *	
2-piperidinone	hydroquinone sulfate *	Xenobiotics
4-vinylphenol sulfate	indolin-2-one	
dihydroferulic acid *	dihydroferulic acid	
O-sulfo-L-tyrosine	quinate *	
quinate	salicylate	

*: Metabolites with an asterisk were determined to have the highest importance to group separation, as calculated by MDA.

**Table 2:** Top 30 plasma metabolites differing between CTRL- and TEST-fed dogs at differing stages of exercise (0 h, 3 h, and 25 h), according to RFA analysis.

CTRL 0 h vs. TEST 0 h	CTRL 3 h vs. TEST 3 h	CTRL 25 h vs. TEST 25 h	Metabolite Super Pathway
3-methylhistidine	3-(4-hydroxyphenyl)propionate	3-methylhistidine *	Amino Acid
homocitrulline	3-hydroxy-3-phenylpropionate	apsaragine	
N-acetyl-3-methylhistidine	4-hydroxyphenylacetyl glycine	homocitrulline *	
p-cresol-sulfate	homocitrulline	imidazole propionate	
3-methoxytyrosine	isovaleryglycine	isobutyrylglycine	
valylleucine	phenylacetylglycine *	isoleucine	Peptide
cyclo(gly pro)	phenylpropionylglycine	N6-acetyllysine	
cis-Cyclo[L-ala-L-Pro]	cis-Cyclo[L-ala-L-Pro]	N-acetyl-3-methylhistidine	
glycylproline	cyclo(gly pro)	o-cresol sulfate	
2-docosahexaenoylglycerophosphocholine *	cyclo(L-phe-L-pro)	p-cresol-sulfate *	
2-hydroxydecanoate	valylleucine	taurine	Carbohydrate
hexanoylglycine	ribonate	cyclo(gly pro)	
docosahexaenoate (DHA; 22:6n3)	1-docosahexaenoylglycerophosphocholine (22:6n3) *	cyclo(L-phe-L-pro)	
alpha-hydroxycaproate	1-docosahexaenoylglycerol	glycylisoleucine	
1-docosahexaenoylglycerophosphocholine (22:6n3)	1-docosahexaenoylglycerophosphoethanolamine	glycylproline	
eicosapentaenoate (EPA; 20:5n3)	2-docosahexaenoylglycerophosphocholine	valylisoleucine	Lipid
1-docosahexaenoylglycerophosphoethanolamine	2-hydroxydecanoate	valylleucine	
1-eicosapentaenoylglycerophosphocholine (20:5n3)	alpha-hydroxyisocaproate	1-docosahexaenoylglycerol	
pyridoxal *	docosahexaenoate (DHA; 22:6n3)	alpha-hydroxycaproate	
1-methylnicotinamide *	propionylglycine	azelate (nonanedioate)	
N1-Methyl-2-pyridone-5-carboxamide	scyllo-inositol	pimelate (heptanedioate) *	Cofactors and Vitamins
alpha-CEHC glucuronide	1-methylnicotinamide *	propionylglycine	
nicotinamide	alpha-CEHC glucuronide *	1-methylnicotinamide *	
2-hydroxyhippurate (salicylurate) *	alpha-CEHC sulfate	alpha-CEHC glucuronide	
4-ethylphenylsulfate *	N1-Methyl-2-pyridone-5-carboxamide	N1-Methyl-2-pyridone-5-carboxamide	
3-methyl catechol sulfate (2)	nicotinamide	pyridoxal	Xenobiotics
3-methyl catechol sulfate (1)	3-methyl catechol sulfate (1) *	pyridoxate	
ectoine	3-methyl catechol sulfate (2)	2-hydroxyhippurate (salicylurate)	
2-aminophenol sulfate	ectoine	3-methyl catechol sulfate (1)	
equol sulfate	equol sulfate	3-methyl catechol sulfate (2)	

*: Metabolites with an asterisk were determined to have the highest importance to group separation, as calculated by MDA.

**Table 3:** Plasma metabolites related to amino acid (leucine/isoleucine/valine, lysine, taurine, urea cycle, creatine, glutathione) or carbohydrate (glycolysis, gluconeogenesis, and pyruvate) metabolism, categorized by sub pathway.

		Fold Change									*p*-Values		
		Ctrl 3 h	Ctrl 25 h	Ctrl 25 h	Test 3 h	Test 25 h	Test 25 h	Test 0 h	Test 3 h	Test 25 h	Diet Main Effect	Time Main Effect	Diet—Time Interaction
Sub Pathway	Metabolite Name	Ctrl 0 h	Ctrl 0 h	Ctrl 3 h	Test 0 h	Test 0 h	Test 3 h	Ctrl 0 h	Ctrl 3 h	Ctrl 25 h			
Leucine, Isoleucine and Valine Metabolism	leucine	1.20	1.13	0.95	1.32	1.21	0.92 *	1.00	1.10	1.07	0.0567	<0.0001	0.3379
	N-acetylleucine	1.39	1.13	0.81	1.31	1.23	0.94	1.27	1.19 *	1.38	0.0071	<0.0001	0.1186
	4-methyl-2-oxopentanoate	1.63	1.04	0.64	1.74	1.11 *	0.64	0.89	0.95	0.95	0.3028	<0.0001	0.7315
	isovalerate	1.03	0.96	0.92	1.03	1.14	1.11	1.14	1.13	1.35	0.0103	0.7937	0.3818
	isovalerylglycine	1.35	1.94	1.44	1.34	1.73	1.30	0.47	0.46	0.42	<0.0001	<0.0001	0.6001
	isovalerylcarnitine	1.08	1.37	1.27	1.17	1.64	1.40	0.89	0.96	1.06	0.8013	<0.0001	0.1948
	beta-hydroxyisovalerate	0.92	1.07	1.16	0.93	1.07	1.16	1.16	1.18	1.17 *	0.0730	0.0034	0.9616
	beta-hydroxyisovaleroylcarnitine	1.01	1.13	1.11	0.90	1.39	1.53	1.45	1.29	1.78 *	0.1113	0.0107	0.4191
	3-methylglutaconate	1.19	1.04	0.87 *	1.11	1.07	0.96	0.68	0.64	0.70	0.0009	0.0417	0.6048
	alpha-hydroxyisovalerate	1.31	1.02	0.78	1.44	1.16	0.80	0.56	0.62	0.64	0.0006	<0.0001	0.3018
	methylsuccinate	0.62	1.02	1.63	0.82	0.95	1.15	0.78	1.02	0.72	0.0068	<0.0001	0.0015
	isoleucine	1.22	1.07	0.88	1.31	1.21	0.93	1.18	1.27	1.34	<0.0001	<0.0001	0.3054
	allo-isoleucine	1.31	0.97	0.74	0.77	1.00	1.30	0.74	0.44	0.76	0.0055	0.9793	0.2081
	N-acetylisoleucine	1.18	1.29	1.10	1.01	1.18	1.17	1.58	1.35	1.44	0.0011	0.0001	0.1594
	3-methyl-2-oxovalerate	1.74	0.99	0.57	1.88	1.23	0.65	1.06	1.14	1.31	0.0389	<0.0001	0.1205
	2-methylbutyrylcarnitine (C5)	1.10	1.22	1.11	0.99	1.58	1.59	1.16	1.04	1.50	0.4132	<0.0001	0.0005
	2-methylbutyrylglycine	1.10	1.65	1.49	1.15	1.34	1.16	0.73	0.76	0.59 *	0.2244	0.0353	0.4448
	tiglyl carnitine	0.98	1.11	1.13	0.55	1.23	2.24	1.59	0.89	1.76	0.4462	0.0205	0.0029
	tigloylglycine	1.21 *	1.76	1.45	1.47	1.51	1.03	0.46	0.55	0.39 *	0.1282	0.0392	0.3210
	2-hydroxy-3-methylvalerate	0.54	0.36	0.67 *	0.98	0.59	0.60	0.54	0.98	0.88	0.1274	0.0002	0.2777
	3-hydroxy-2-ethylpropionate	1.31	1.12 *	0.86	1.16	1.18	1.01	1.10	0.98	1.16 *	0.3311	<0.0001	0.0675
	ethylmalonate	1.16	1.14	0.98	1.12	1.04	0.93 *	0.88	0.85 *	0.80	0.0664	0.0004	0.2254
	valine	1.20	1.18	0.98	1.23	1.25	1.01	1.04	1.07	1.10	0.0207	<0.0001	0.6248
	N-acetylvaline	1.33	1.35	1.01	1.12	1.22	1.08 *	1.03	0.87 *	0.93	0.3970	<0.0001	0.0314
	3-methyl-2-oxobutyrate	1.55	1.09	0.70	1.69	1.24	0.73	0.91	0.99	1.03	0.6597	<0.0001	0.5646
	isobutyrylcarnitine	1.07	1.40	1.31	1.00	1.64	1.63	1.06	1.00	1.24	0.6007	<0.0001	0.0444
	isobutyrylglycine	1.14	1.78	1.56	0.91	1.52	1.66	0.48	0.39	0.41	<0.0001	<0.0001	0.5134
	3-hydroxyisobutyrate	1.36	1.29	0.95	1.40	1.39	1.00	1.00	1.03	1.08	0.4510	<0.0001	0.4424
	alpha-hydroxyisocaproate	0.80	0.53	0.67	1.14	0.71	0.63	0.48	0.68	0.63	0.0011	<0.0001	0.0511
Lysine Metabolism	lysine	1.61	1.25	0.78	1.50	1.47	0.98	1.34	1.25	1.58	<0.0001	<0.0001	0.0228
	N2-acetyllysine	0.72	1.18	1.64	1.09	1.14	1.05	0.56	0.85 *	0.54	0.0008	0.0004	0.0020
	N6-acetyllysine	0.94	1.48	1.58	0.98	1.17 *	1.20	0.52	0.54	0.41	<0.0001	<0.0001	0.0400
	N-6-trimethyllysine	0.95	1.19	1.25	0.90	1.29	1.44	1.15 *	1.09	1.25	0.0404	<0.0001	0.3009
	2-aminoadipate	1.29	1.30	1.00	1.02	1.32	1.30	1.00	0.78	1.02	0.3958	<0.0001	0.0059
	glutarate (pentanedioate)	0.27	0.70	2.60	0.15	0.53	3.59	1.03	0.57	0.78	0.0119	<0.0001	0.0005
	glutarylcarnitine (C5)	1.30	1.16	0.89	1.26	1.27	1.01	1.96	1.89	2.15	0.1303	0.0001	0.9342
	3-methylglutarylcarnitine (1)	2.18	2.14	0.98	1.62	2.25	1.39	1.62	1.20	1.71	0.0274	<0.0001	0.0509
	pipecolate	0.77	0.90	1.18	0.84	0.98	1.17	0.67	0.73	0.73	<0.0001	0.0001	0.7182
Methionine, Cysteine, SAM and Taurine Metabolism	methionine	0.97	1.05	1.08	1.10 *	1.30	1.18	1.24	1.42	1.54	<0.0001	0.0004	0.0251
	N-acetylmethionine	1.21	1.04	0.86	1.07	1.11	1.04	1.08	0.95	1.14	0.5633	0.0301	0.1488
	methionine sulfone	1.10 *	1.13	1.03	1.40	1.40	1.00	1.09	1.39	1.35	0.2204	<0.0001	0.0027
	methionine sulfoxide	0.96	1.33	1.39	1.27	1.64	1.28	1.03	1.37	1.27	0.0038	<0.0001	0.1143
	S-adenosylhomocysteine (SAH)	1.03	0.76	0.74	1.18	1.03	0.88	0.83	0.94	1.12	0.9948	0.0479	0.7515
	alpha-ketobutyrate	3.64	1.05	0.29	3.35	1.44	0.43	1.00	0.92	1.37	0.7566	<0.0001	0.3626
	2-aminobutyrate	2.51	1.00	0.40	1.56	1.13	0.72	1.58	0.98	1.77	0.0002	<0.0001	0.0035
	2-hydroxybutyrate	4.07	0.98	0.24	2.39	1.07	0.45	1.41 *	0.83	1.53	0.2397	<0.0001	0.0058
	cystine	0.79 *	0.86	1.10	0.70	0.75 *	1.08	1.33	1.18	1.16	0.0118	0.0105	0.7740
	S-methylcysteine	1.31	1.02	0.78	1.20	0.94	0.78	0.89	0.81	0.82	0.0179	0.0067	0.6816
	hypotaurine	1.00	1.00	1.00	1.63	1.59	0.98	1.02	1.66	1.62	0.0089	0.0464	0.0464
	taurine	1.48	1.20	0.81	1.77	1.24 *	0.70	2.55	3.04	2.65	<0.0001	<0.0001	0.3118
	N-acetyltaurine	0.88	1.00	1.13	0.92	0.99	1.07	0.98	1.03	0.97	0.7659	<0.0001	0.5565
Urea cycle; Arginine and Proline Metabolism	arginine	1.09 *	1.17	1.08 *	1.15	1.17	1.02	1.13 *	1.20	1.14 *	0.0284	<0.0001	0.6794
	urea	0.81	1.14 *	1.41	0.74	1.11*	1.50	1.25	1.14	1.22 *	0.0517	<0.0001	0.5822
	ornithine	1.44	1.25	0.87	1.77	1.40	0.79 *	0.88	1.08	0.99	0.8512	<0.0001	0.6379
	proline	0.97	1.17	1.21	1.12	1.34	1.20	0.83	0.96	0.95	0.1012	<0.0001	0.2578
	citrulline	0.92	0.98	1.06	0.99	1.08 *	1.09 *	0.79	0.85	0.88	0.0993	0.0659	0.1748
	homoarginine	4.14	0.90	0.22	1.47	0.87	0.59	5.33	1.90	5.15	0.0001	0.0004	0.0375
	homocitrulline	0.80	1.04	1.30	0.91	0.90	0.98	0.10	0.12	0.09	<0.0001	<0.0001	0.0109
	dimethylarginine (SDMA + ADMA)	1.28	0.98	0.77	1.33	1.01	0.75	0.85	0.88	0.87	0.0072	<0.0001	0.6859
	N-acetylarginine	1.15	1.31	1.14	1.61	1.33	0.82 *	0.86	1.21 *	0.88	0.5934	0.0001	0.0287
	N-delta-acetylornithine	0.81	1.03	1.27	0.91	0.98	1.08	0.57	0.64	0.54	0.0001	<0.0001	0.0036
	N-methyl proline	0.93 *	1.03	1.11	1.06	1.01	0.95 *	0.44	0.51	0.43	0.0007	0.5746	0.0044
	trans-4-hydroxyproline	0.71	1.27	1.78	0.91	1.62	1.77	0.68	0.87	0.87	0.0063	<0.0001	0.2023
	pro-hydroxy-pro	1.90	1.97	1.04	1.97	2.33	1.18	1.19	1.23	1.4 *	0.1079	<0.0001	0.7146
Creatinine Metabolism	creatine	2.38	1.51	0.63	3.36	1.78	0.53	1.17	1.65	1.37	0.0313	<0.0001	0.0031
	creatinine	0.96	1.15	1.20	1.06	1.12	1.05	1.00	1.11	0.97	0.8284	<0.0001	0.0381
	creatine phosphate	1.06	0.60	0.57	0.99	0.78	0.79	0.96	0.90	1.25	0.6166	0.0018	0.2288
	guanidinoacetate	0.97	1.20	1.23	0.95	1.29	1.36	1.04	1.01	1.12	0.7870	<0.0001	0.3922
Glutathione Metabolism	glutathione, oxidized (GSSG)	1.37	1.02	0.75	2.59	1.35 *	0.52	0.61 *	1.15	0.80	0.2919	<0.0001	0.0503
	cysteine-glutathione disulfide	1.18	0.95	0.80	1.40	1.04	0.74	0.90	1.07	0.99	0.7721	<0.0001	0.2921
	S-methylglutathione	1.38	1.10	0.79	1.52	1.07	0.70	0.91	1.00	0.89	0.3568	<0.0001	0.1914
	5-oxoproline	1.31	0.95	0.73	1.27	0.99	0.78	0.91	0.88 *	0.95	0.0581	<0.0001	0.7234
	ophthalmate	5.29	1.30	0.25	7.46	1.62	0.22	0.39	0.55 *	0.48	0.0054	<0.0001	0.5956
Glycolysis, Gluconeogenesis, and Pyruvate Metabolism	1,5-anhydroglucitol (1,5-AG)	1.01	1.04	1.03	1.03	1.06	1.03	0.82	0.83	0.83	0.1539	0.0017	0.7695
	glucose	1.21	0.95	0.78	1.27	0.98	0.77	0.88	0.93	0.91	0.0260	0.0007	0.9644
	pyruvate	2.35	1.32 *	0.56	2.96	1.42	0.48	0.67	0.84	0.72	0.0699	<0.0001	0.4201
	lactate	1.62	1.21	0.75	1.71	1.15 *	0.67	0.86	0.91	0.82 *	0.0825	<0.0001	0.5738
	glycerate	1.30	1.13	0.87	1.29	1.12	0.87	0.92	0.92	0.92	0.0914	<0.0001	0.9710

Numbers in red were significantly (*p* < 0.05) higher for that comparison, whereas numbers in green were lower. Numbers with an asterisk (*) at the end reached statistical trends (0.05 < *p* < 0.10).

**Table 4:** Plasma metabolites related to cofactor and vitamin metabolism, categorized by sub pathway.

		Fold Change									*p*-Values		
		Ctrl 3 h	Ctrl 25 h	Ctrl 25 h	Test 3 h	Test 25 h	Test 25 h	Test 0 h	Test 3 h	Test 25 h	Diet Main Effect	Time Main Effect	Diet—Time Interaction
Sub Pathway	Metabolite Name	Ctrl 0 h	Ctrl 0 h	Ctrl 3 h	Test 0 h	Test 0 h	Test 3 h	Ctrl 0 h	Ctrl 3 h	Ctrl 25 h			
Nicotinate and Nicotinamide Metabolism	quinolinate	0.98	0.92	0.94	1.66	1.46	0.88	0.73	1.24	1.16	0.8132	0.5277	0.3803
	nicotinamide	0.73	1.05	1.45	1.02	1.31	1.29	7.21	10.03	8.95	<0.0001	0.2315	0.2809
	1-methylnicotinamide	1.34	6.47	4.84	0.88	1.57 *	1.78	37.75	24.82	9.14	<0.0001	<0.0001	0.0004
	trigonelline (N’-methylnicotinate)	1.17	1.43	1.23	1.48	1.53	1.03	1.78	2.27	1.9	<0.0001	0.0003	0.3944
	N1-Methyl-2-pyridone-5-carboxamide	0.82	1.6	1.96	0.41	0.96	2.32	8.63	4.37	5.17	<0.0001	<0.0001	0.0010
Riboflavin Metabolism	riboflavin (vitamin B2)	1.37	0.87	0.63	1.27	1.35	1.06	1.15	1.07	1.78	0.1600	0.0283	0.0256
	flavin adenine dinucleotide (FAD)	1.36	1.52	1.11	1.2	1.28	1.07	1.33	1.17	1.12	0.4174	0.4811	0.8184
Pantothenate and CoA Metabolism	pantothenate	0.64	0.52	0.82	0.78	0.78	0.99	2.4	2.92	3.56	0.0006	<0.0001	0.0098
Ascorbate and Aldarate Metabolism	threonate	1.06	0.95	0.89	1.06	0.97	0.92	0.99	0.99	1.02	0.8897	0.0006	0.8163
	oxalate (ethanedioate)	1.07	0.95	0.89	1.08	0.98	0.91	0.94	0.95	0.97	0.4052	<0.0001	0.8343
	gulonic acid *	0.94 *	0.92	0.97	0.89	0.9	1	0.85	0.81	0.83	0.1641	0.0009	0.7309
Tocopherol Metabolism	alpha-tocopherol	1.1	0.95	0.86	0.77	0.84 *	1.09	1.56	1.09	1.38	0.0053	0.3178	0.0866
	gamma-tocopherol	1.06	1.04	0.98	1.09	2.15	1.96	1.16	1.2	2.4	0.0226	0.0462	0.0467
	gamma-CEHC glucuronide *	1	1	1	0.31	2.4	7.84	5.87	1.8 *	14.07	<0.0001	<0.0001	<0.0001
	alpha-CEHC glucuronide *	0.44 *	0.34	0.76	0.5	0.98	1.97	11.55	13.04	33.67	<0.0001	0.0039	0.0223
	alpha-CEHC sulfate	0.38	0.37	0.96	0.62	1.19	1.92 *	4.21	6.83	13.58	<0.0001	0.2066	0.3825
	alpha-CEHC	0.37	0.47	1.26	0.22	0.67	3.08	18.79	11.07	26.97	<0.0001	0.0019	0.0392
Biotin Metabolism	biotin	0.53	0.13	0.25 *	0.31	0.75 *	2.38 *	3.6	2.14	20.59	0.0002	0.0038	0.0145
Tetrahydrobiopterin Metabolism	biopterin	1.14	0.81 *	0.71	1.22 *	0.98	0.81	0.87	0.93	1.06	0.6628	0.0008	0.6454
	dihydrobiopterin	1.11	1.01	0.92	1.01	0.96	0.95	1	0.91	0.95	0.6464	0.2632	0.7898
Pterin Metabolism	pterin	3.48	1.48 *	0.43	3.7	1.39	0.38	0.88	0.93	0.83	0.3144	<0.0001	0.8634
Hemoglobin and Porphyrin Metabolism	heme	0.54	0.34	0.64	0.58	0.27	0.47	0.71	0.77	0.57	0.3130	0.3135	0.5558
Vitamin B6 Metabolism	pyridoxal	0.65 *	0.71	1.09	0.54	1.23	2.25	9.76	8.13	16.84	<0.0001	0.0006	0.0386
	pyridoxate	0.87	0.98	1.12	0.62	1.56	2.5	2.44	1.75	3.9	<0.0001	0.0002	0.0031

Metabolite names with an asterisk (*) at the end indicate that these compound identities were not officially confirmed based on a standard, but have high confidence in identification. Numbers in red were significantly (*p* < 0.05) higher for that comparison, whereas numbers in green were lower. Numbers with an asterisk (*) at the end reached statistical trends (0.05 < *p* < 0.10).

**Table 5:** Plasma metabolites related to lipid metabolism, categorized by sub pathway.

		Fold Change									*p*-Values		
		Ctrl 3 h	Ctrl 25 h	Ctrl 25 h	Test 3 h	Test 25 h	Test 25 h	Test 0 h	Test 3 h	Test 25 h	Diet Main Effect	Time Main Effect	Diet—Time Interaction
Sub Pathway	Metabolite Name	Ctrl 0 h	Ctrl 0 h	Ctrl 3 h	Test 0 h	Test 0 h	Test 3 h	Ctrl 0 h	Ctrl 3 h	Ctrl 25 h			
Fatty Acid, Dicarboxylate	pimelate (heptanedioate)	1.04	1.45	1.40	1.22 *	1.34	1.09	0.49	0.58	0.45	<0.0001	<0.0001	0.2088
	suberate (octanedioate)	0.86	1.23	1.44	0.94	1.15	1.22 *	0.61	0.67	0.57	<0.0001	0.0017	0.3504
	azelate (nonanedioate)	1.30	1.56	1.19	1.66	1.71	1.03	0.41	0.52	0.45	<0.0001	<0.0001	0.6097
	sebacate (decanedioate)	1.08	0.91	0.84	0.79	1.26 *	1.60	0.70	0.51	0.98	0.0036	0.1932	0.0220
	eicosanodioate	0.80	1.03	1.28	0.95	1.09	1.15	0.67	0.78	0.70	0.0014	0.0001	0.0918
Fatty Acid, Amino	2-aminoheptanoate	0.51	0.98	1.93	0.56	1.09 *	1.94	0.31	0.34	0.34	<0.0001	<0.0001	0.3391
	2-aminooctanoate	1.35	1.28	0.95	0.88	1.01	1.15	0.62	0.40	0.49	0.0002	0.5898	0.1888
Fatty Acid Metabolism (also BCAA Metabolism)	butyrylglycine	0.94	2.09	2.22	2.52	1.65	0.65	0.17	0.44	0.13	<0.0001	0.6205	0.2341
	propionylglycine	0.84	1.16	1.38	1.01	1.30	1.28	0.41	0.49	0.46	<0.0001	0.0615	0.7149
Fatty Acid Metabolism (Acyl Glycine)	hexanoylglycine	0.75	1.26 *	1.68	0.98	1.37	1.40	0.34	0.44	0.36	<0.0001	<0.0001	0.1188
	N-palmitoyl glycine	0.90	0.98	1.08	0.92	0.87	0.95	0.64	0.66	0.57	0.0002	0.3062	0.5765
	N-linoleoylglycine	0.93	0.86	0.92	1.22	1.23	1.01	0.29	0.38	0.42	<0.0001	0.8233	0.9692
Carnitine Metabolism	deoxycarnitine	0.92	1.30	1.42	1.12	1.31	1.17	0.80	0.98	0.80 *	0.1576	<0.0001	0.0012
Fatty Acid, Monohydroxy	alpha-hydroxycaproate	1.27 *	1.11	0.88	1.72	1.45	0.84	0.23	0.30	0.29	<0.0001	0.0007	0.3776
	2-hydroxyoctanoate	1.10	1.26	1.14	1.13	1.07	0.95	0.54	0.55	0.46	<0.0001	0.2033	0.5113
	2-hydroxydecanoate	1.25 *	0.86	0.69	1.17	0.73	0.62	0.48	0.44	0.40	<0.0001	<0.0001	0.6251
	2-hydroxypalmitate	1.09	0.97	0.89	1.09	0.99	0.91	0.79	0.79	0.81	0.0072	0.1711	0.9687
	2-hydroxystearate	1.00	0.93	0.93	0.91	0.87	0.95	0.86	0.78	0.80	0.0099	0.0731	0.5576
	3-hydroxypropanoate	0.54	0.44	0.80	0.88	0.86	0.97	0.39	0.62	0.75	<0.0001	0.0376	0.0847
	13-HODE + 9-HODE	1.19	1.13	0.95	1.02	1.13	1.11	0.42	0.36	0.42	0.0003	0.8163	0.8916
Sterol	cholesterol	1.02	0.95	0.93	1.08	1.07	0.99	0.71	0.75	0.80	0.0023	0.8118	0.6388
Primary Bile Acid Metabolism	taurocholate	1.51	2.41	1.60 *	1.70 *	2.02	1.19	0.51	0.58 *	0.43	0.0007	0.0031	0.4881
	chenodeoxycholate	0.47	1.04	2.22	0.58	1.14	1.96	0.32	0.40	0.35	0.0002	0.1112	0.8612
	taurochenodeoxycholate	1.66	2.34	1.41	2.05	1.68	0.82	0.42	0.52	0.30	<0.0001	0.0039	0.2147
Secondary Bile Acid Metabolism	taurodeoxycholate	1.44 *	2.26	1.57	1.45	1.86 *	1.28	0.66	0.66	0.54	0.0295	0.0020	0.5292
	taurolithocholate	1.11	0.97	0.87	1.39	1.84	1.32	0.33	0.42	0.63	0.0118	0.3025	0.8285
	tauroursodeoxycholate	1.11	1.74 *	1.56	1.27	2.06	1.62	0.25	0.29	0.30	<0.0001	0.0107	0.6574
Polyunsaturated Fatty Acid (n3 and n6)	stearidonate (18:4n3)	1.12	0.51	0.46	1.31	0.97	0.74	2.16	2.54	4.11	<0.0001	0.0001	0.0352
	eicosapentaenoate (EPA; 20:5n3)	0.86	0.63	0.73	0.80 *	0.94	1.17	2.73	2.55	4.05	<0.0001	0.0278	0.0448
	docosapentaenoate (n3 DPA; 22:5n3)	1.17	0.84	0.72 *	1.10	0.94	0.85	1.30	1.22	1.45	0.0120	0.2202	0.7518
	docosahexaenoate (DHA; 22:6n3)	1.16	1.01	0.87	1.01	1.05	1.04	3.60	3.13	3.73	<0.0001	0.6405	0.5714
Lysolipid	1-eicosapentaenoylglycerophosphocholine (20:5n3) *	0.66	1.02	1.56	0.42	0.80	1.88	3.40	2.20	2.65	<0.0001	<0.0001	0.1693
	1-docosapentaenoylglycerophosphocholine (22:5n3) *	1.31	1.25 *	0.96	1.07	0.93	0.87	1.65	1.35 *	1.23	0.0163	0.1630	0.1928
	2-docosapentaenoylglycerophosphocholine (22:5n3) *	0.94	1.20	1.27 *	1.02	0.81	0.79 *	1.77	1.92	1.19	0.0114	0.9517	0.0347
	1-docosahexaenoylglycerophosphocholine (22:6n3) *	1.22	1.49	1.22	0.96	0.98	1.02	5.24	4.11	3.44	<0.0001	0.1331	0.1230
	2-docosahexaenoylglycerophosphocholine *	1.07	1.52	1.43	0.81	0.89	1.10	4.96	3.78	2.90	<0.0001	0.1520	0.1906
	1-palmitoylglycerophosphoethanolamine	1.20	1.37	1.14	1.05	1.20 *	1.14	1.54	1.34	1.35	0.0024	0.0092	0.6970
	2-palmitoylglycerophosphoethanolamine *	1.50	1.73	1.15	1.00	1.02	1.02	2.07	1.38	1.22	0.0292	0.0674	0.1686
	2-docosahexaenoylglycerophosphoethanolamine *	0.70	1.58 *	2.25	0.59 *	0.83	1.41	4.25	3.56	2.22 *	0.0022	0.0153	0.1836
	2-eicosapentaenoylglycerophosphoethanolamine *	1.13	0.83	0.74	0.35	1.06	3.03	7.85	2.44	10.01	<0.0001	0.1362	0.0533
	1-docosahexaenoylglycerophosphoethanolamine *	1.22 *	0.82	0.67	0.80	0.67	0.84	4.03	2.65	3.30	<0.0001	0.0057	0.0604
	1-stearoylglycerophosphoglycerol	1.33	1.21	0.92	0.75	0.30	0.41	2.57	1.45	0.64	0.3329	0.0566	0.0236
Sphingolipid Metabolism	palmitoyl sphingomyelin	1.04	1.03	0.99	1.10	0.99	0.90	1.13	1.20	1.08	0.0018	0.0839	0.2724
	nervonoyl sphingomyelin *	0.95	0.89	0.94	1.23	1.00	0.81	1.38	1.79	1.56	<0.0001	0.1829	0.0720
	palmitoleoyl sphingomyelin *	1.10	1.01	0.92	1.14	1.02	0.90 *	1.09	1.13	1.11	0.0325	0.0151	0.8269

Metabolite names with an asterisk (*) at the end indicate that these compound identities were not officially confirmed based on a standard, but have high confidence in identification. Numbers in red were significantly (*p* < 0.05) higher for that comparison, whereas numbers in green were lower. Numbers with an asterisk (*) at the end reached statistical trends (0.05 < *p* < 0.10).

## Data Availability

Additional data are available upon reasonable request to the corresponding author.

## Supplementary Materials

The following supporting information can be downloaded at https://www.mdpi.com/article/10.3390/metabo16060397/s1: Table S1: Ingredient and chemical composition and guaranteed analysis of standard performance diet (control) and nutrient-fortified endurance dog food (test); Table S2–S4: Metabolites differing at baseline determined by ANOVA contrasts, separated by lipid metabolism (Table S2), AA metabolism (Table S3), and peptide, carbohydrate, nucleotide, cofactors and vitamins, and xenobiotic metabolism (Table S4); Table S5: Plasma metabolites related to lipid metabolism (Table S5) and xenobiotic metabolism (Table S6); Figure S1–S5: Top 30 serum metabolites that differed in dogs consuming CTRL at 0 h versus CTRL at 3 h and CTRL at 25 h (Figure S1); TEST at 0 h versus TEST at 3 h and TEST at 25 h (Figure S2); CTRL at 0 h versus TEST at 0 h (Figure S3); CTRL at 3 h versus TEST at 3 h (Figure S4); CTRL at 25 h versus TEST at 25 h (Figure S5).

## Abbreviations

The following abbreviations are used in this manuscript:

AA	Amino acids
BA	Bile acids
BCAA	Branched-chain amino acids
CTRL	Standard performance diet
FA	Fatty acids
GC-MS	Gas chromatography-mass spectrometry
GSH	Reduced glutathione
GSSG	Oxidized glutathione
LIMS	Laboratory Information Management System
*m*/*z*	Mass to charge ratio
RFA	Randon forest analysis
RI	Retention time/index
ROS	Reactive oxygen species
RSD	Relative standard deviation
TEST	High endurance formula
UPLC-MS/MS	Ultrahigh performance liquid chromatography-tandem mass spectrometry
