# Supplementary material for: Plasma Metabolite Profiles of Exercising American Foxhound Dogs Fed Different Diets
Source: Metabolites. 2026 Jun 8;16(6):397. doi: 10.3390/metabo16060397 (PMC13303894; doi:10.3390/metabo16060397)

Supplementary Table S1. Nutrient analysis and ingredient composition of standard performance diet (control; CTRL) and nutrient-fortified endurance dog food (test; TEST)

Item	CTRL[1]	TEST[2]
Dry Matter (DM), %	94.1	92.2
	%, DM basis	
Crude protein, %	28.1	32.9
Acid hydrolyzed fat, %	21.8	23.6
Crude fiber, %	2.4	2.2
Ash, %	10.3	12.3
Nitrogen Free Extract, %[3]	31.0	35.4
Metabolizable energy[4], kcal/kg	4,269	4,466
Lysine, %	1.4	2.2
Methionine, %	0.5	0.9
Cystine, %	0.4	0.4
Leucine, %	2.0	2.3
Isoleucine, %	1.1	1.4
Valine, %	1.4	1.6
Arginine, %	1.7	2.2
Histidine	0.6	0.8
Phenylalanine, %	1.1	1.3
Threonine, %	1.1	1.4
Tryptophan, %	0.3	0.4
Linoleic acid (18:1,%)	3.9	4.4
Zinc, mg/kg	147	338
Vitamin E (tocopherols), IU/kg	86	1,507
Vitamin C (L-ascorbyl-2-phosphate), mg/100g	2.3	35.1
Lutein, ug/g	1.4	5.9
Taurine, %	0.1	0.3

[1]Standard commercial performance diet; Chicken by-product meal, ground yellow corn, meat meal, ground wheat, chicken fat (preserved with mixed tocopherols), dried beet pulp, fish meal, flaxseed, salt, vitamin A supplement, vitamin D3 supplement, choline chloride, vitamin B12 supplement, folic acid, thiamine mononitrate, pyridoxine hydrochloride, biotin, calcium iodate, copper sulfate, ferrous sulfate, manganous oxide, zinc oxide, magnesium oxide.

[2]NUTRO® NATURAL CHOICE® High Endurance Chicken Meal Whole Brown Rice & Oatmeal Formula; The Nutro Company, Franklin, TN, USA; Chicken meal, whole brown rice, rice bran, chicken fat (preserved with mixed tocopherols), whole grain oatmeal, pea protein, brewers rice, chicken, dried plain beet pulp, natural flavor, fish oil (preserved with mixed tocopherols), potassium chloride, salt, choline chloride, vitamin E supplement, sunflower oil (preserved with mixed tocopherols), soybean oil (preserved with mixed tocopherols), taurine, DL-methionine, zinc sulfate, L-ascorbyl-2-polyphosphate (source of vitamin C), biotin, niacin supplement, calcium pantothenate, riboflavin supplement (vitamin B2), pyridoxine hydrochloride (vitamin B6), copper proteinate, iron proteinate, selenium yeast, vitamin B12 supplement, L-carnitine, manganese proteinate, potassium iodide, vitamin

A supplement, thiamine mononitrate (vitamin B1), vitamin D3 supplement, folic acid, rosemary extract, decaffeinated green tea extract, spearmint extract.

[3]Calculated values, as NFE = 100 – (crude protein + acid hydrolyzed fat + crude fiber + ash).

[4]ME calculated based on equation described at NRC (2006).

Supplementary Table S2. Lipid metabolism metabolites differing at baseline determined by ANOVA contrasts, separated by sub pathways

**Sub Pathway**	**Metabolite Name**	**Test 0h / Ctrl 0h**	**ANOVA Contrast p-value**
Medium Chain Fatty Acid	laurate (12:0)	**0.71**	0.0011
Long Chain Fatty Acid	arachidate (20:0)	**0.77**	0.0041
Polyunsaturated Fatty Acid (n3 and n6)	stearidonate (18:4n3)	**2.16**	0.0001
	eicosapentaenoate (EPA; 20:5n3)	**2.73**	<0.0001
	docosahexaenoate (DHA; 22:6n3)	**3.6**	<0.0001
	linolenate [alpha or gamma; (18:3n3 or 6)]	**0.56**	0.0134
Fatty Acid, Branched	13-methylmyristic acid	**0.77**	0.0263
	15-methylpalmitate (isobar with 2-methylpalmitate)	**0.72**	0.0047
Fatty Acid, Dicarboxylate	pimelate (heptanedioate)	**0.49**	<0.0001
	suberate (octanedioate)	**0.61**	<0.0001
	azelate (nonanedioate)	**0.41**	<0.0001
	sebacate (decanedioate)	**0.7**	0.0340
	eicosanodioate	**0.67**	0.0003
Fatty Acid, Amino	2-aminoheptanoate	**0.31**	<0.0001
	2-aminooctanoate	**0.62**	0.0340
Fatty Acid Metabolism (also BCAA Metabolism)	butyrylglycine	**0.17**	0.0006
	propionylglycine	**0.41**	<0.0001
Fatty Acid Metabolism(Acyl Glycine)	hexanoylglycine	**0.34**	<0.0001
	N-palmitoyl glycine	**0.64**	0.0013
	N-linoleoylglycine	**0.29**	<0.0001
Carnitine Metabolism	deoxycarnitine	**0.8**	0.0466
Fatty Acid, Monohydroxy	4-hydroxybutyrate (GHB)	**1.4**	0.0335
	alpha-hydroxycaproate	**0.23**	<0.0001
	2-hydroxyoctanoate	**0.54**	<0.0001
	2-hydroxydecanoate	**0.48**	<0.0001
	2-hydroxypalmitate	**0.79**	0.0208
	3-hydroxypropanoate	**0.39**	<0.0001
	13-HODE + 9-HODE	**0.42**	0.0095
Endocannabinoid	oleic ethanolamide	**0.69**	0.0448
	palmitoyl ethanolamide	**0.64**	0.0015
Inositol Metabolism	scyllo-inositol	**0.35**	<0.0001
Lysolipid	1-eicosapentaenoylglycerophosphocholine (20:5n3)*	**3.4**	<0.0001
	1-docosapentaenoylglycerophosphocholine (22:5n3)*	**1.65**	0.0028
	2-docosapentaenoylglycerophosphocholine (22:5n3)*	**1.77**	0.0129
	1-docosahexaenoylglycerophosphocholine (22:6n3)*	**5.24**	<0.0001
	2-docosahexaenoylglycerophosphocholine*	**4.96**	<0.0001
	1-palmitoylglycerophosphoethanolamine	**1.54**	0.0031

	2-palmitoylglycerophosphoethanolamine*	2.07	0.0046
	2-arachidonoylglycerophosphoethanolamine*	0.61	0.0145
	2-docosahexaenoylglycerophosphoethanolamine*	4.25	0.0006
	2-eicosapentaenoylglycerophosphoethanolamine*	7.85	<0.0001
	1-docosahexaenoylglycerophosphoethanolamine*	4.03	<0.0001
	1-palmitoylglycerophosphoglycerol*	1.94	0.0410
	1-stearoylglycerophosphoglycerol	2.57	0.0415
	palmitoyl-arachidonoyl-glycerophosphocholine (1)*	0.9	0.0212
	palmitoyl-arachidonoyl-glycerophosphocholine (2)*	0.88	0.0043
	palmitoyl-linoleoyl-glycerophosphocholine (1)*	0.87	0.0290
	palmitoyl-linoleoyl-glycerophosphocholine (2)*	0.85	0.0047
	stearoyl-arachidonoyl-glycerophosphocholine (1)*	0.87	0.0466
	stearoyl-arachidonoyl-glycerophosphoinositol (1)*	0.78	0.0052
	oleoyl-linoleoyl-glycerophosphocholine (1)*	0.81	0.0005
	oleoyl-linoleoyl-glycerophosphocholine (2)*	0.84	0.0062
	stearoyl-linoleoyl-glycerophosphocholine (1)*	0.75	<0.0001
	stearoyl-linoleoyl-glycerophosphocholine (2)*	0.78	0.0002
Monoacylglycerol	1-stearoylglycerol (1-monostearin)	0.75	0.0154
	1-oleoylglycerol (1-monoolein)	0.71	0.0313
	1-linoleoylglycerol (1-monolinolein)	0.75	0.0076
	2-linoleoylglycerol (2-monolinolein)	0.74	0.0342
	1-docosahexaenoylglycerol	3.89	<0.0001
Sphingolipid Metabolism	palmitoyl sphingomyelin	1.13	0.0198
	myristoyl sphingomyelin*	0.82	0.0187
	nervonoyl sphingomyelin*	1.38	0.0061
Sterol	cholesterol	0.71	0.0023
Primary Bile Acid Metabolism	taurocholate	0.51	0.0178
	chenodeoxycholate	0.32	0.0019
	taurochenodeoxycholate	0.42	0.0009
Secondary Bile Acid Metabolism	taurolithocholate	0.33	0.0295
	tauroursodeoxycholate	0.25	<0.0001

Metabolite names with an asterisk (*) at the end indicate that these compound identities were not officially confirmed based on a standard, but have high confidence in identification.

Numbers in red were significantly ($p<0.05$) higher for that comparison, whereas numbers in green were lower.

Supplementary Table S3. Amino acid metabolism metabolites differing at baseline determined by ANOVA contrasts, separated by sub pathways

Sub Pathway	Metabolite Name	Test 0h / Ctrl 0h	ANOVA Contrast p-value
Glycine, Serine and Threonine Metabolism	glycine	0.76	0.0128
	N-acetylglycine	0.47	<0.0001
Alanine and Aspartate Metabolism	asparagine	1.33	0.0014
Glutamate Metabolism	glutamine	0.84	0.0104
	N-acetylglutamate	0.79	0.0356
Histidine Metabolism	N-acetylhistidine	1.25	0.0138
	3-methylhistidine	2.66	<0.0001
	N-acetyl-3-methylhistidine*	3.77	<0.0001
	imidazole propionate	0.54	<0.0001
	imidazole lactate	1.93	0.0001
Lysine Metabolism	lysine	1.34	0.0002
	N2-acetyllysine	0.56	0.0003
	N6-acetyllysine	0.52	<0.0001
	3-methylglutarylcarnitine (1)	1.62	0.0096
	pipecolate	0.67	<0.0001
Phenylalanine and Tyrosine Metabolism	phenyllactate (PLA)	0.55	0.0048
	4-hydroxyphenylacetate	0.57	0.0108
	phenylacetylglycine	0.68	0.0126
	tyrosine	0.83	0.0164
	3-(4-hydroxyphenyl)lactate	0.74	0.0036
	p-cresol sulfate	0.29	<0.0001
	o-cresol sulfate	0.43	0.0045
	3-methoxytyrosine	0.6	<0.0001
	phenylpropionylglycine	0.37	0.0082
	4-hydroxyphenylacetyl glycine	0.33	<0.0001
Leucine, Isoleucine and Valine Metabolism	N-acetylleucine	1.27	0.0132
	isovalerylglycine	0.47	<0.0001
	3-methylglutaconate	0.68	0.0032
	alpha-hydroxyisovalerate	0.56	0.0002
	methylsuccinate	0.78	0.0031
	isoleucine	1.18	0.0023
	N-acetylisoleucine	1.58	0.0003
	2-hydroxy-3-methylvalerate	0.54	0.0303
	isobutyrylglycine	0.48	<0.0001
	alpha-hydroxyisocaproate	0.48	<0.0001
Methionine, Cysteine, SAM and Taurine Metabolism	methionine	1.24	0.0044
	2-aminobutyrate	1.58	0.0002

	cystine	1.33	0.0369
	taurine	2.55	<0.0001
Urea cycle; Arginine and Proline Metabolism	urea	1.25	0.0498
	proline	0.83	0.0205
	citrulline	0.79	0.0433
	homoarginine	5.33	<0.0001
	homocitrulline	0.1	<0.0001
	dimethylarginine (SDMA + ADMA)	0.85	0.0061
	N-delta-acetylornithine	0.57	<0.0001
	N-methyl proline	0.44	0.0005
	trans-4-hydroxyproline	0.68	0.0012
Polyamine Metabolism	N-acetylputrescine	0.76	0.0083
Guanidino and Acetamido Metabolism	4-guanidinobutanoate	1.69	0.0154
Glutathione Metabolism	ophthalmate	0.39	0.0070

Metabolite names with an asterisk (*) at the end indicate that these compound identities were not officially confirmed based on a standard, but have high confidence in identification.

Numbers in red were significantly ($p<0.05$) higher for that comparison, whereas numbers in green were lower.

Supplementary Table S4. Metabolites differing at baseline determined by ANOVA contrasts, separated by super and sub pathways

Super Pathway	Sub Pathway	Metabolite Name	Test 0h / Ctrl 0h	ANOVA Contrast p-value
Peptide	Gamma-glutamyl Amino Acid	gamma-glutamylglutamine	**0.83**	0.0123
		gamma-glutamylleucine	**0.87**	0.0468
		gamma-glutamyltyrosine	**0.72**	0.0007
		gamma-glutamylvaline	**0.84**	0.0374
		gamma-glutamyl-2-aminobutyrate	**1.33**	0.0179
	Dipeptide Derivative	N-acetylcarnosine	**0.61**	0.0003
	Dipeptide	aspartylleucine	**0.77**	0.0192
		aspartylphenylalanine	**0.58**	0.0117
		cyclo(gly-pro)	**0.21**	<0.0001
		cyclo(leu-pro)	**0.61**	0.0069
		cyclo(L-phe-D-pro)*	**0.13**	<0.0001
		cyclo(L-phe-L-pro)	**0.19**	0.0050
		glycylisoleucine	**0.41**	0.0020
		glycylleucine	**0.38**	<0.0001
		glycylproline	**0.09**	<0.0001
		glycylvaline	**0.72**	0.0147
		prolylserine	**0.55**	0.0077
		pyroglutamylvaline	**0.75**	0.0490
		valylisoleucine	**0.29**	<0.0001
		valylleucine	**0.05**	<0.0001
		cis-Cyclo[L-ala-L-Pro]	**0.34**	<0.0001
Carbohydrate	Glycolysis, Gluconeogenesis, and Pyruvate Metabolism	pyruvate	**0.67**	0.0466
	Fructose, Mannose and Galactose Metabolism	fructose	**0.73**	0.0388
		mannitol	**0.14**	<0.0001
		galactonate	**2.2**	0.0096
	Aminosugar Metabolism	glucuronate	**0.71**	0.0011
Nucleotide	Purine Metabolism, Guanine containing	7-methylguanine	**0.9**	0.0334
	Pyrimidine Metabolism, Uracil containing	uracil	**1.68**	0.0299
		2'-deoxyuridine	**1.48**	0.0134
Cofactors and Vitamins	Nicotinate and Nicotinamide Metabolism	nicotinamide	**7.21**	<0.0001
		1-methylnicotinamide	**37.75**	<0.0001
		trigonelline (N'-methylnicotinate)	**1.78**	0.0005
		N1-Methyl-2-pyridone-5-carboxamide	**8.63**	<0.0001
	Pantothenate and CoA Metabolism	pantothenate	**2.4**	0.0035
	Tocopherol Metabolism	alpha-tocopherol	**1.56**	0.0008

		gamma-CEHC glucuronide*	**5.87**	<0.0001
		alpha-CEHC glucuronide*	**11.55**	<0.0001
		alpha-CEHC sulfate	**4.21**	<0.0001
		alpha-CEHC	**18.79**	<0.0001
	Biotin Metabolism	biotin	**3.6**	0.0001
	Vitamin B6 Metabolism	pyridoxal	**9.76**	<0.0001
		pyridoxate	**2.44**	<0.0001
Xenobiotics	Benzoate Metabolism	2-hydroxyhippurate (salicylurate)	**6.86**	<0.0001
		3-methyl catechol sulfate (1)	**0.1**	<0.0001
		3-methyl catechol sulfate (2)	**0.27**	<0.0001
		4-methylcatechol sulfate	**0.38**	0.0086
		4-ethylphenylsulfate	**0.17**	<0.0001
		4-vinylphenol sulfate	**1.81**	0.0009
	Food Component/Plant	2,3-dihydroxyisovalerate	**0.64**	0.0044
		equol sulfate	**0.2**	<0.0001
		erythritol	**0.62**	0.0003
		homostachydrine*	**0.26**	<0.0001
		quinate	**1.57**	0.0453
		S-propylcysteine	**0.44**	<0.0001
		stachydrine	**0.71**	0.0004
		4-allylphenol sulfate	**0.46**	0.0001
		methyl glucopyranoside (alpha + beta)	**0.81**	0.0117
	Drug	4-acetylphenol sulfate	**0.53**	0.0007
		6-oxopiperidine-2-carboxylic acid	**0.79**	0.0364
		pivaloylcarnitine	**1.37**	0.0395
		salicylate	**3.97**	<0.0001
	Chemical	2-aminophenol sulfate	**4.71**	<0.0001
		2-hydroxyisobutyrate	**2.27**	0.0381
		S-(3-hydroxypropyl)mercapturic acid (HPMA)	**0.29**	0.0048
		ectoine	**4.19**	<0.0001
		phenylcarnitine*	**0.41**	0.0099

Metabolite names with an asterisk (*) at the end indicate that these compound identities were not officially confirmed based on a standard, but have high confidence in identification.

Numbers in red were significantly ($p<0.05$) higher for that comparison, whereas numbers in green were lower.

Supplementary Table S5. Plasma metabolites related to lipid metabolism, categorized by sub pathway

		Fold Change									*p*-values		
Sub Pathway	Metabolite Name	Ctrl 3h / Ctrl 0h	Ctrl 25h / Ctrl 0h	Ctrl 25h / Ctrl 3h	Test 3h / Test 0h	Test 25h / Test 0h	Test 25h / Test 3h	Test 0h / Ctrl 0h	Test 3h / Ctrl 3h	Test 25h / Ctrl 25h	Diet Main Effect	Time Main Effect	Diet : Time Interaction
Medium Chain Fatty Acid	caprate (10:0)	**1.20**	**0.77**	**0.64**	1.00	0.93	0.93	**0.86***	**0.72**	1.05	0.0098	<0.0001	0.0007
	laurate (12:0)	1.06	**0.74**	**0.70**	1.08	1.04	0.97	**0.71**	**0.73**	1.01	0.0051	0.0061	0.0135
Long Chain Fatty Acid	palmitate (16:0)	1.14	**0.72**	**0.63**	0.91	**0.81**	0.89	1.01	**0.80**	1.12	0.5809	<0.0001	0.0351
	margarate (17:0)	1.11	**0.64**	**0.58**	0.95	**0.78**	**0.82***	0.78	**0.66**	0.95	0.0159	<0.0001	0.1062
	stearate (18:0)	1.09	**0.87**	**0.80**	0.94	**0.86**	0.92	0.93	**0.80**	0.92	0.0144	0.0005	0.1141
	arachidate (20:0)	1.16	0.86	**0.74**	1.08	1.01	0.94	**0.77**	**0.72**	0.91	0.0004	0.0325	0.2243
Polyunsaturated Fatty Acid (n3 and n6)	docosatrienoate (22:3n3)	1.09	**0.52**	**0.48**	0.93	0.91	0.97	0.83	**0.71***	1.44	0.5989	0.1540	0.0429
	linolenate [alpha or gamma; (18:3n3 or 6)]	1.05	**0.46**	**0.44**	0.97	**0.78**	**0.80***	**0.56**	**0.52**	0.94	0.0060	<0.0001	0.1192
	dihomo-linolenate (20:3n3 or n6)	1.03	**0.81**	**0.78**	0.88	**0.85***	0.96	0.86	**0.74**	0.91	0.0128	0.0239	0.3072
	arachidonate (20:4n6)	1.05	1.15	1.10	0.90	0.96	1.06	0.92	**0.79**	**0.77**	0.0085	0.4595	0.5028
Fatty Acid, Branched	13-methylmyristic acid	1.12	**0.68**	**0.61**	1.11	0.92	0.83	**0.77**	**0.76**	1.03	0.0318	0.0011	0.1427
	15-methylpalmitate (isobar with 2-methylpalmitate)	1.15	**0.63**	**0.55**	1.04	0.85	**0.82***	**0.72**	**0.65**	0.97	0.0010	<0.0001	0.0525
Fatty Acid, Dicarboxylate	undecanedioate	**0.43***	**0.27**	**0.62**	**0.31**	**0.39**	1.26	1.59	1.14	**2.32**	0.2084	<0.0001	0.0328
	dodecanedioate	**0.50**	**0.29**	**0.58**	**0.26**	**0.58**	**2.23**	1.29	0.67	**2.59**	0.2861	<0.0001	<0.0001
	hexadecanedioate	0.97	**0.70**	**0.73**	0.91	0.94	1.04	1.14	1.07	**1.53**	0.3788	0.1992	0.0390
Fatty Acid Metabolism (also BCAA Metabolism)	propionylcarnitine	**1.48**	**1.40**	0.94	**1.45**	**2.28**	**1.58**	0.88	0.86	**1.44**	0.8257	<0.0001	0.0090
Fatty Acid, Monohydroxy	4-hydroxybutyrate (GHB)	0.88	0.85	0.97	0.96	1.07	1.12	**1.40**	**1.52**	**1.76**	0.0054	0.6548	0.9598
	16-hydroxypalmitate	0.94	**0.43**	**0.46**	0.90	0.77	0.85	1.02	0.99	**1.84***	0.5915	0.0024	0.0198
Endocannabinoid	oleic ethanolamide	0.78	**0.67**	0.86	0.81	**0.76**	0.93	**0.69**	**0.72**	**0.78***	0.0059	0.0083	0.9038
	palmitoyl ethanolamide	0.91	0.94	1.03	1.22	1.11	0.91	**0.64**	0.85	**0.75**	0.0010	0.8838	0.3353
Inositol Metabolism	scyllo-inositol	**1.36***	1.32	0.97	**1.34***	**1.49**	1.11	**0.35**	**0.35**	**0.40**	<0.0001	0.0086	0.8275
	inositol 1-phosphate (I1P)	0.96	1.11	1.16	**0.73**	0.93	**1.28***	0.84	**0.64**	**0.70**	0.0180	0.1469	0.3551
	myo-inositol	**1.43**	**1.29**	0.90	**1.63**	**1.39**	0.85	1.10	**1.25***	1.19	0.0296	<0.0001	0.7329
Phospholipid Metabolism	choline	**1.18**	**1.33**	**1.13**	**1.28**	**1.21**	0.94	0.99	1.07	**0.90***	0.6576	<0.0001	0.0487

	glycerophosphorylcholine (GPC)	0.93	1.03	1.11	0.87	0.92	1.06	0.80	0.75	**0.71***	0.0123	0.5876	0.9530
	glycerophosphoethanolamine	0.93	1.09	**1.17***	**0.83**	0.96	**1.16***	**0.86***	**0.77**	**0.76**	0.0013	0.0276	0.6655
Lysolipid	1-linoleoylglycerophosphocholine (18:2n6)	**0.73**	1.09	**1.49**	**0.67**	0.87	**1.30**	0.90	0.83	**0.72**	0.0489	<0.0001	0.2727
	1-linolenoylglycerophosphocholine (18:3n3)*	**0.44**	1.11	**2.52**	**0.41**	**0.73**	**1.79**	0.74	**0.69***	**0.49**	0.0124	<0.0001	0.1830
	2-linolenoylglycerophosphocholine(18:3n3)*	**0.43**	1.17	**2.71**	**0.36**	0.79	**2.16**	0.73	**0.62**	**0.49**	0.0181	<0.0001	0.4302
	1-docosapentaenoylglycerophosphocholine (22:5n6)*	**1.75**	1.64	0.94	0.75	0.75	1.00	0.86	**0.37**	**0.40**	0.0046	0.6496	0.0667
	1-palmitoylplasmenylethanolamine*	0.66	1.13	**1.72**	**0.47**	**0.38**	0.81	1.38	0.98	**0.47**	0.2976	0.0122	0.0178
	1-stearoylplasmenylethanolamine*	0.92	0.92	1.01	0.86	0.91	1.06	**0.78***	**0.73**	**0.77***	0.0069	0.4140	0.9486
	1-linoleoylglycerophosphoethanolamine*	0.99	**1.38**	**1.38**	**0.73**	0.85	1.17	0.98	**0.72**	**0.61**	0.0093	0.0377	0.0972
	2-linoleoylglycerophosphoethanolamine*	0.98	1.33	**1.36***	**0.69**	0.84	1.22	0.94	**0.67**	**0.60**	0.0155	0.0715	0.2991
	1-arachidonoylglycerophosphoethanolamine*	**1.32**	1.10	**0.83***	0.96	**0.81***	0.84	0.84	**0.61**	**0.62**	0.0002	0.0713	0.0831
	2-arachidonoylglycerophosphoethanolamine*	0.95	1.21	**1.28***	1.18	1.03	0.87	**0.61**	0.75	**0.51**	0.0058	0.5149	0.2710
	1-stearoylglycerophosphoinositol	0.83	1.19	1.42	**0.47**	**0.51**	1.10	1.09	**0.61**	**0.47**	0.0034	0.0032	0.0159
	2-stearoylglycerophosphoinositol*	0.92	1.13	1.23	**0.50**	**0.55**	1.10	1.05	**0.57**	**0.51**	0.0085	0.0157	0.0452
	1-linoleoylglycerophosphoinositol*	**0.62**	1.00	**1.63**	**0.43**	**0.70**	**1.64**	0.96	**0.67**	**0.67**	0.0198	<0.0001	0.2119
	2-linoleoylglycerophosphoinositol*	**0.66**	1.09	**1.66**	**0.38**	0.69	**1.83**	1.00	**0.57**	**0.63**	0.0185	<0.0001	0.2822
	1-arachidonoylglycerophosphoinositol*	0.83	0.97	1.17	**0.55**	**0.64**	1.16	0.93	**0.62**	**0.62**	0.0025	0.0052	0.1665
	2-arachidonoylglycerophosphoinositol*	0.89	1.08	1.22	**0.46**	**0.58**	**1.25***	1.04	**0.54**	**0.56**	0.0017	0.0017	0.0154
	palmitoyl-arachidonoyl-glycerophosphocholine (1)*	0.96	1.04	**1.09**	**0.94***	**1.06***	**1.12**	**0.90**	**0.88**	**0.91**	0.0071	0.0005	0.8114
	palmitoyl-arachidonoyl-glycerophosphocholine (2)*	0.97	**1.09**	**1.13**	0.95	**1.06***	**1.11**	**0.88**	**0.87**	**0.86**	0.0005	<0.0001	0.8719
	palmitoyl-linoleoyl-glycerophosphocholine (1)*	**0.77**	1.05	**1.37**	**0.80**	1.06	**1.33**	**0.87**	0.91	**0.88**	0.0207	<0.0001	0.8144

	palmitoyl-linoleoyl-glycerophosphocholine (2)*	0.75	1.07	1.43	0.80	1.09	1.36	0.85	0.91*	0.86	0.0065	<0.0001	0.5080
	stearoyl-arachidonoyl-glycerophosphocholine (1)*	1.28	1.13	0.88	1.29	1.15	0.89	0.87	0.87*	0.88*	0.0422	<0.0001	0.9649
	stearoyl-arachidonoyl-glycerophosphocholine (2)*	1.32	1.21	0.92	1.26	1.15	0.91	0.88*	0.85	0.84	0.0295	<0.0001	0.4898
	stearoyl-arachidonoyl-glycerophosphoinositol (1)*	0.91	1.00	1.10	0.89*	0.97	1.09	0.78	0.76	0.76	0.0007	0.0826	0.9543
	oleoyl-linoleoyl-glycerophosphocholine (1)*	0.72	0.92	1.28	0.73	0.96	1.30	0.81	0.83	0.84	0.0005	<0.0001	0.7923
	oleoyl-linoleoyl-glycerophosphocholine (2)*	0.73	0.89	1.23	0.72	0.94	1.30	0.84	0.83	0.88	0.0051	<0.0001	0.5566
	stearoyl-linoleoyl-glycerophosphocholine (1)*	0.78	0.98	1.25	0.82	1.02	1.25	0.75	0.78	0.78	<0.0001	<0.0001	0.5487
	stearoyl-linoleoyl-glycerophosphocholine (2)*	0.81	0.98	1.21	0.83	1.00	1.21	0.78	0.79	0.79	0.0001	<0.0001	0.9172
Glycerolipid Metabolism	glycerol	0.96	0.95	0.99	0.92	0.90	0.98	0.80	0.76	0.75	0.0128	0.6321	0.7284
Monoacylglycerol	1-myristoylglycerol (1-monomyristin)	0.96	1.15	1.20	0.77	0.64	0.83	1.01	0.81	0.56	0.0514	0.3389	0.0146
	1-palmitoylglycerol (1-monopalmitin)	1.44*	1.27	0.88	1.59	1.29*	0.81	0.77*	0.85	0.78*	0.0161	0.0072	0.7628
	1-stearoylglycerol (1-monostearin)	1.35	1.16	0.86	1.25*	1.10	0.88	0.75	0.69	0.71	0.0001	0.0333	0.9273
	1-oleoylglycerol (1-monoolein)	1.09	1.09	1.00	1.23	1.10	0.90	0.71	0.80	0.71	0.0014	0.4666	0.8688
	2-oleoylglycerol (2-monoolein)	1.04	1.32	1.27	0.96	0.95	0.98	0.83	0.77*	0.60	0.0014	0.7244	0.3441
	1-linoleoylglycerol (1-monolinolein)	0.87	1.03	1.19	0.78*	0.92	1.17	0.75	0.68	0.67	<0.0001	0.0683	0.8008
	2-linoleoylglycerol (2-monolinolein)	0.66	0.87	1.31*	0.66	0.80*	1.22	0.74	0.74	0.69	0.0006	0.0004	0.8916
	1-arachidonylglycerol	0.94	1.01	1.08	0.77*	0.78	1.02	0.76*	0.62	0.58	0.0007	0.2262	0.4250
	2-arachidonoyl glycerol	0.60	0.95	1.59	0.60	0.46	0.78	0.87	0.86	0.42	0.0047	0.0007	0.0045
	1-docosahexaenoylglycerol	0.83	0.95	1.15	0.74*	0.70	0.94	3.89	3.46	2.84	<0.0001	0.1713	0.2173
Sphingolipid Metabolism	myristoyl sphingomyelin*	1.06	1.03	0.98	1.10	1.10	1.00	0.82	0.85	0.87*	0.0031	0.3434	0.8817

Metabolite names with an asterisk (*) at the end indicate that these compound identities were not officially confirmed based on a standard, but have high confidence in identification.

Numbers in red were significantly (p<0.05) higher for that comparison, whereas numbers in green were lower. Fold change values with an asterisk (*) at the end reached statistical trends (0.05<p<0.10).

Supplementary Table S6. Plasma metabolites related to xenobiotic metabolism, categorized by sub pathway

		Fold Change									*p*-values		
**Sub Pathway**	**Metabolite Name**	**Ctrl 3h / Ctrl 0h**	**Ctrl 25h / Ctrl 0h**	**Ctrl 25h / Ctrl 3h**	**Test 3h / Test 0h**	**Test 25h / Test 0h**	**Test 25h / Test 3h**	**Test 0h / Ctrl 0h**	**Test 3h / Ctrl 3h**	**Test 25h / Ctrl 25h**	**Diet Main Effect**	**Time Main Effect**	**Diet : Time Interaction**
Benzoate Metabolism	hippurate	0.47	1.13	2.39	0.41	0.53	1.31	0.66*	0.57	0.31	0.0013	0.0003	0.1661
	2-hydroxyhippurate (salicylurate)	0.52	0.71	1.36	0.10	0.47	4.56	6.86	1.35	4.53	0.0003	<0.0001	0.0005
	4-hydroxyhippurate	0.47	2.02*	4.29	0.33	0.35	1.08	1.23	0.85	0.21	0.1360	0.0009	0.0026
	4-hydroxymandelate	1.15	0.99	0.86	1.22	1.00	0.82	0.93	0.99	0.95	0.3993	<0.0001	0.4817
	benzoate	0.60	0.80	1.34*	0.41	0.71	1.73	0.66*	0.45	0.58	0.0006	<0.0001	0.1228
	catechol sulfate	0.28	0.59	2.09	0.17	0.38	2.24	0.70	0.42	0.45	0.0001	<0.0001	0.0621
	O-methylcatechol sulfate	0.73	0.93	1.27*	0.45	0.65	1.45	0.91	0.56	0.64	0.0262	<0.0001	0.0054
	3-methyl catechol sulfate (1)	1.38	1.47	1.07	1.81	1.31	0.73	0.10	0.13	0.09	<0.0001	0.0083	0.3666
	3-methyl catechol sulfate (2)	1.36	1.77	1.31	1.00	1.00	1.00	0.27	0.20	0.15	<0.0001	0.0082	0.0082
	4-methylcatechol sulfate	1.89	0.81	0.43	2.34	0.74	0.32	0.38	0.47	0.35	0.0008	0.0126	0.7488
	4-ethylphenylsulfate	0.86	1.05	1.22	1.98	1.15	0.58	0.17	0.39	0.19	<0.0001	0.5482	0.0061
	4-vinylphenol sulfate	0.60	0.59	0.98	0.39	0.50	1.27	1.81	1.18	1.53	0.0154	<0.0001	0.0038
Food Component/Plant	2-piperidinone	0.19	0.87	4.56	0.08	0.50	6.31	1.14	0.48	0.66	0.0896	<0.0001	0.0009
	2,3-dihydroxyisovalerate	1.21	1.69	1.39	2.30	2.14	0.93	0.64	1.21	0.81*	0.0568	<0.0001	0.0224
	2-oxindole-3-acetate	3.74	1.71	0.46	1.23	0.88	0.71	0.85	0.28	0.44	0.0053	0.0360	0.0672
	3-hydroxyindolin-2-one	0.20	0.55	2.80	0.17	0.77*	4.55	0.72	0.61*	0.99	0.1755	<0.0001	0.4972
	gluconate	1.60	1.29	0.81	1.53	1.27	0.83*	1.04	1.00	1.03	0.8910	<0.0001	0.9910
	5-ketogluconate	1.13	0.91	0.81	1.13	0.83*	0.73	0.79*	0.79*	0.71	0.0172	0.0247	0.8021
	cinnamate	0.10	0.29	2.96	0.06	0.26	4.65	0.88	0.50	0.78	0.3436	<0.0001	0.7689
	cinnamoylglycine	0.18	0.50	2.74	0.05	0.31	6.35	1.12	0.30	0.70	0.0118	<0.0001	0.0957
	dihydroferulic acid	0.05	0.53	10.54	0.05	0.57*	11.14	0.86	0.87	0.92	0.2623	<0.0001	0.4819
	equol sulfate	0.61	0.74	1.22	0.40	0.40	1.01*	0.20	0.13	0.11	<0.0001	<0.0001	0.0071
	ergothioneine	0.70	1.21*	1.75	0.97	1.34	1.38	0.76*	1.06	0.84	0.3425	<0.0001	0.0407
	erythritol	1.10	0.90	0.82	1.36	1.06	0.78*	0.62	0.77	0.73	0.0002	0.0249	0.5340

	ferulate	1.39	2.53	1.82	2.26*	2.72	1.20	0.57	0.93	0.62*	0.0256	0.0005	0.7568
	ferulic acid 4-sulfate	1.24	1.80	1.45	1.81	1.69	0.94	0.78	1.13	0.73*	0.1268	0.0002	0.3320
	homostachydrine*	1.05	1.19	1.14	1.41	1.31	0.93	0.26	0.35	0.28	<0.0001	0.0002	0.0173
	indoleacrylate	0.76	0.65	0.85	0.65	0.63	0.98	0.53*	0.45	0.52	0.0273	<0.0001	0.5447
	indolin-2-one	0.20	0.62	3.13	0.14	0.68*	4.89	0.98	0.68*	1.06	0.5839	<0.0001	0.1361
	quinate	23.09	2.41	0.10	31.23	3.06	0.10	1.57	2.12	2.00	0.0025	<0.0001	0.9227
	S-propylcysteine	0.94	0.91	0.96	1.11	1.07	0.96	0.44	0.52	0.52	0.0002	0.9514	0.4721
	stachydrine	0.73	1.04	1.42	0.81	0.96	1.18	0.71	0.79	0.66	0.0004	<0.0001	0.0060
	4-allylphenol sulfate	0.96	1.04	1.08	1.02	1.03	1.01	0.46	0.49	0.45	0.0002	0.1279	0.3033
Drug	4-acetylphenol sulfate	0.70	1.02	1.45	0.60	0.87	1.44	0.53	0.46	0.46	<0.0001	0.0005	0.6132
	6-oxopiperidine-2-carboxylic acid	1.35	1.56	1.16*	1.03	1.07	1.04	0.79	0.60	0.54	<0.0001	0.0007	0.0117
	hydroquinone sulfate	0.61	0.92	1.52	0.30	0.41	1.36	1.40	0.69	0.62	0.8267	<0.0001	0.0004
	pivaloylcarnitine	0.80	0.94	1.17	0.73	0.55	0.76*	1.37	1.24	0.80	0.4024	0.3584	0.0297
	salicylate	0.55	0.66*	1.19	0.16	0.53	3.24	3.97	1.17	3.21	<0.0001	<0.0001	<0.0001
Chemical	sulfate*	0.97	1.12	1.15	1.02	1.13	1.11	1.00	1.05	1.01	0.7037	<0.0001	0.2459
	O-sulfo-L-tyrosine	0.99	1.50	1.51	0.92	1.44	1.57	0.86	0.79	0.82*	0.0279	<0.0001	0.5716
	2-aminophenol sulfate	1.29	0.61*	0.47	0.25	1.17	4.73	4.71	0.90	9.01	<0.0001	<0.0001	<0.0001
	2-hydroxyisobutyrate	1.21	1.18	0.98	1.31	1.19	0.90	2.27	2.47	2.29	0.0240	0.0189	0.7233
	S-(3-hydroxypropyl)mercapturic acid (HPMA)	0.58	0.32	0.54	0.96	0.92	0.95	0.29	0.47	0.83	0.1538	<0.0001	<0.0001
	ectoine	1.29	1.35	1.05	0.85	0.74	0.88	4.19	2.76	2.31	<0.0001	0.8742	<0.0001
	phenylcarnitine*	0.59	1.01	1.70	0.76	1.48	1.94	0.41	0.52	0.60	0.0076	0.0001	0.8101
	N-methylpipecolate	7.29	2.15*	0.29	5.37	1.73*	0.32	0.65	0.48	0.53	0.1681	<0.0001	0.9751

Metabolite names with an asterisk (*) at the end indicate that these compound identities were not officially confirmed based on a standard, but have high confidence in identification.

Numbers in red were significantly ($p<0.05$) higher for that comparison, whereas numbers in green were lower. Fold change values with an asterisk (*) at the end reached statistical trends ($0.05<p<0.10$).

Supplementary Figure S1. Top 30 serum metabolites that differed in dogs consuming CTRL at 0h versus CTRL at 3h and CTRL at 25h, according to random forest analysis.

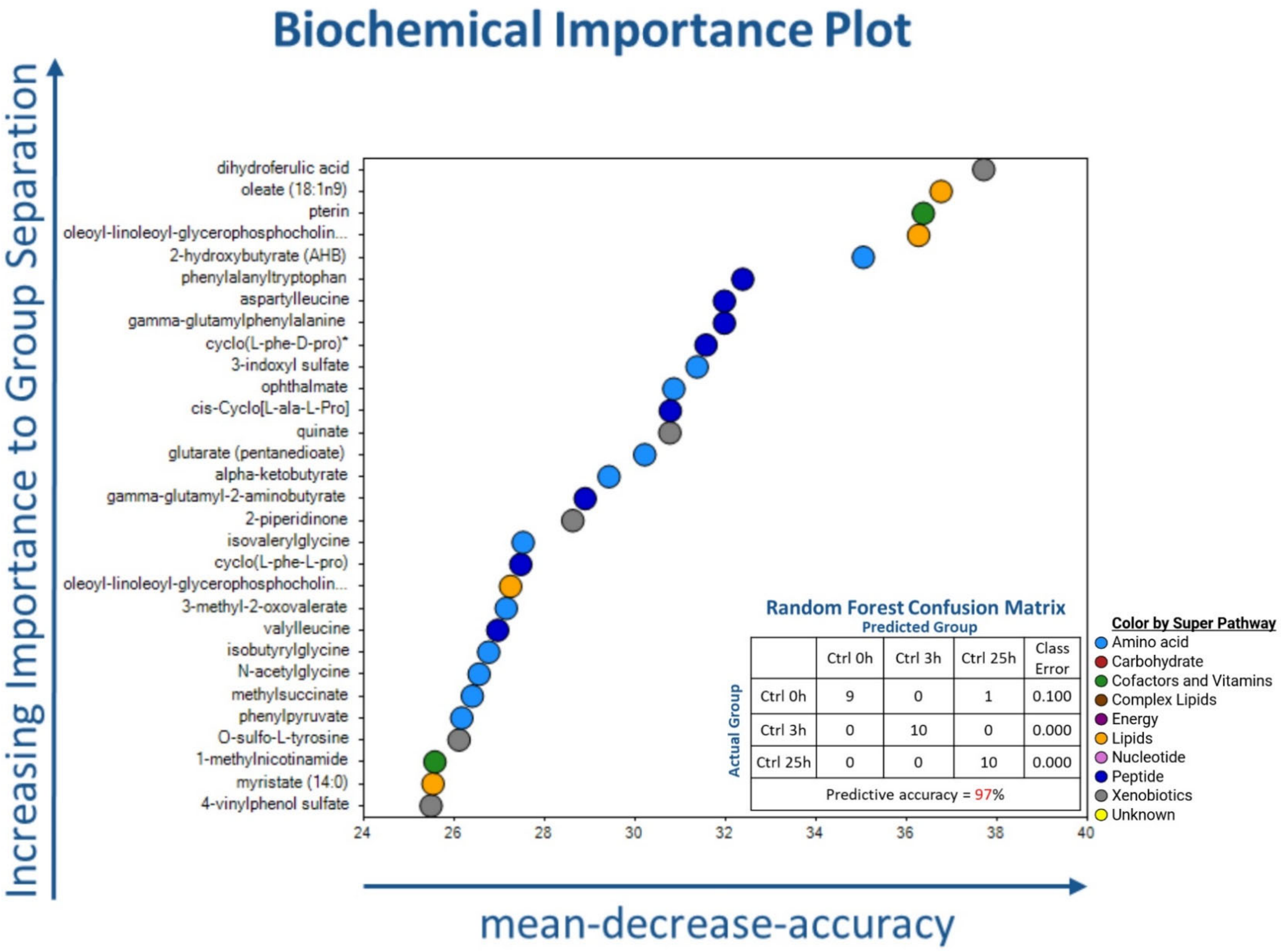

Supplementary Figure S2. Top 30 serum metabolites that differed in dogs consuming TEST at 0h versus TEST at 3h and TEST at 25h, according to random forest analysis.

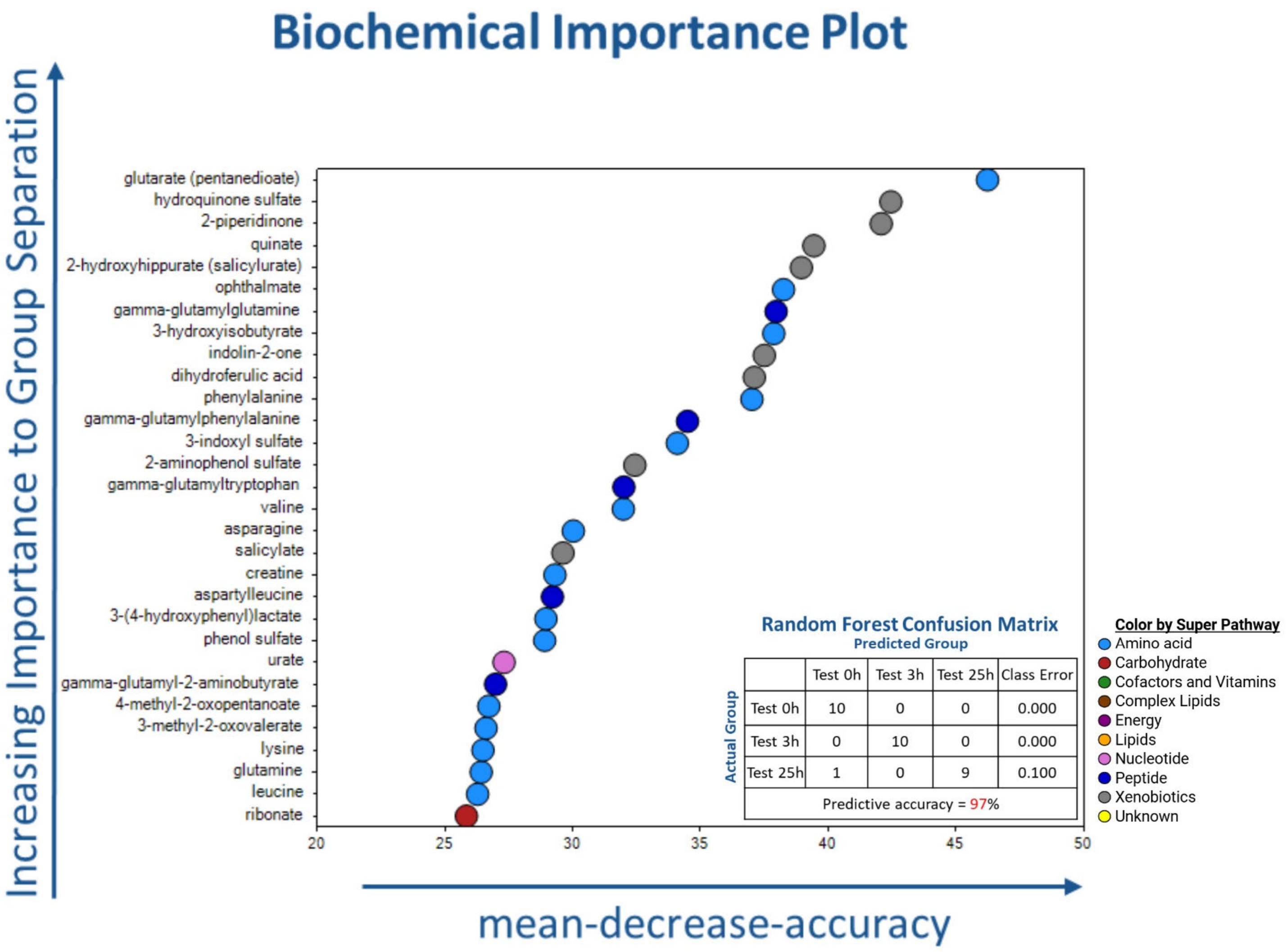

Supplementary Figure S3. Top 30 serum metabolites that differed in dogs consuming CTRL at 0h versus TEST at 0h, according to random forest analysis.

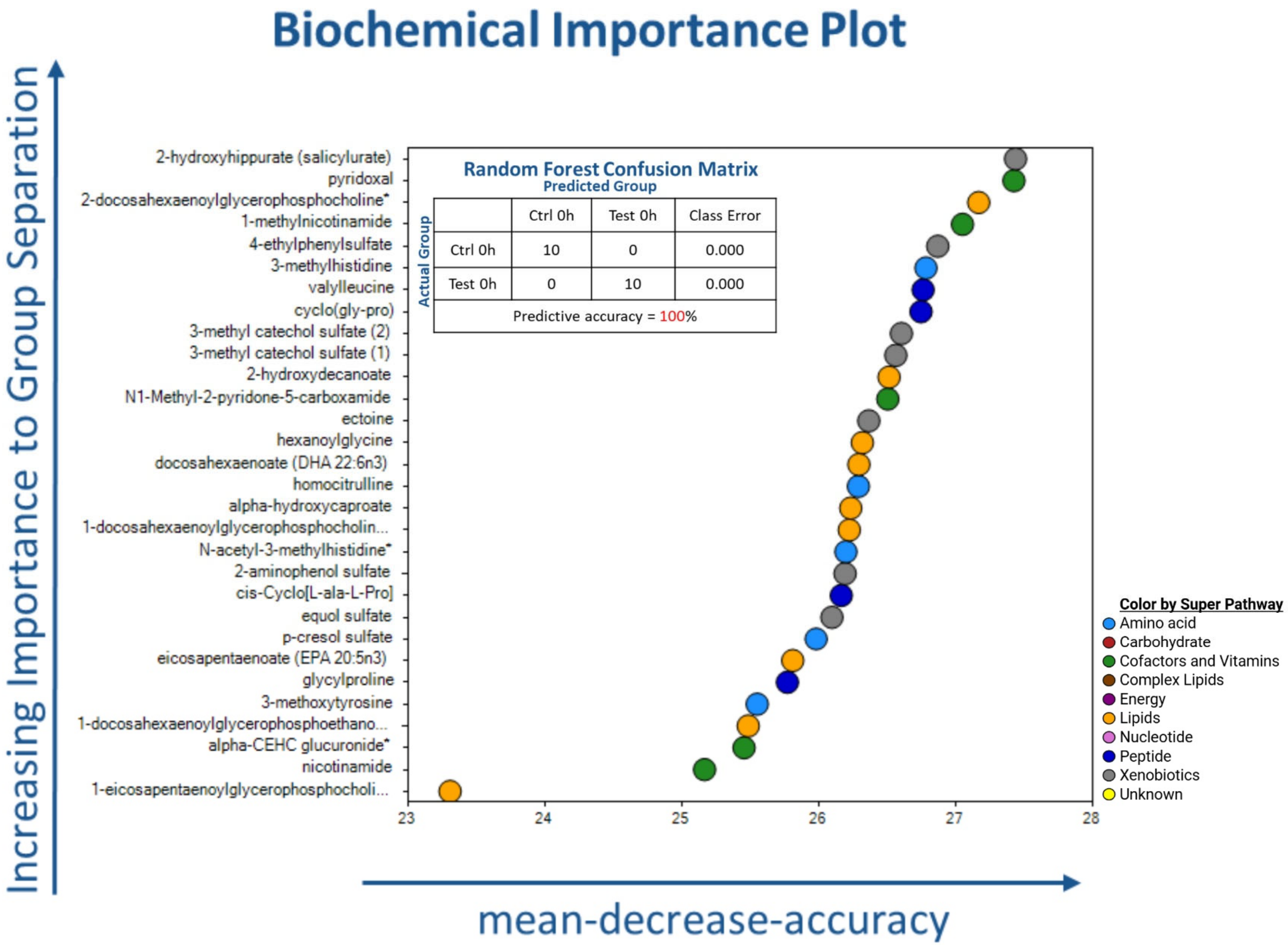

Supplementary Figure S4. Top 30 serum metabolites that differed in dogs consuming CTRL at 3h versus TEST at 3h, according to random forest analysis.

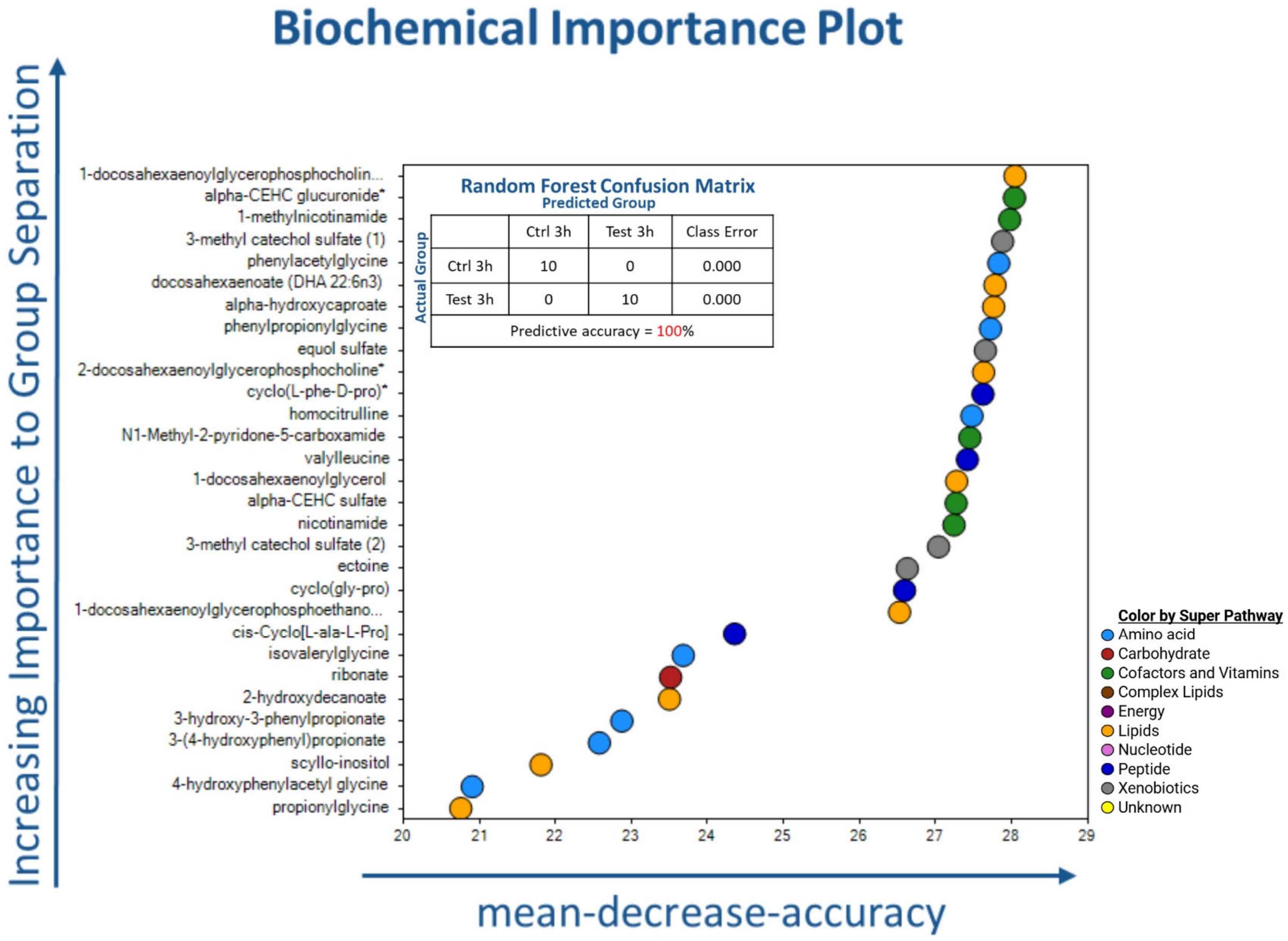

Supplementary Figure S5. Top 30 serum metabolites that differed in dogs consuming CTRL at 25h versus TEST at 25h, according to random forest analysis.

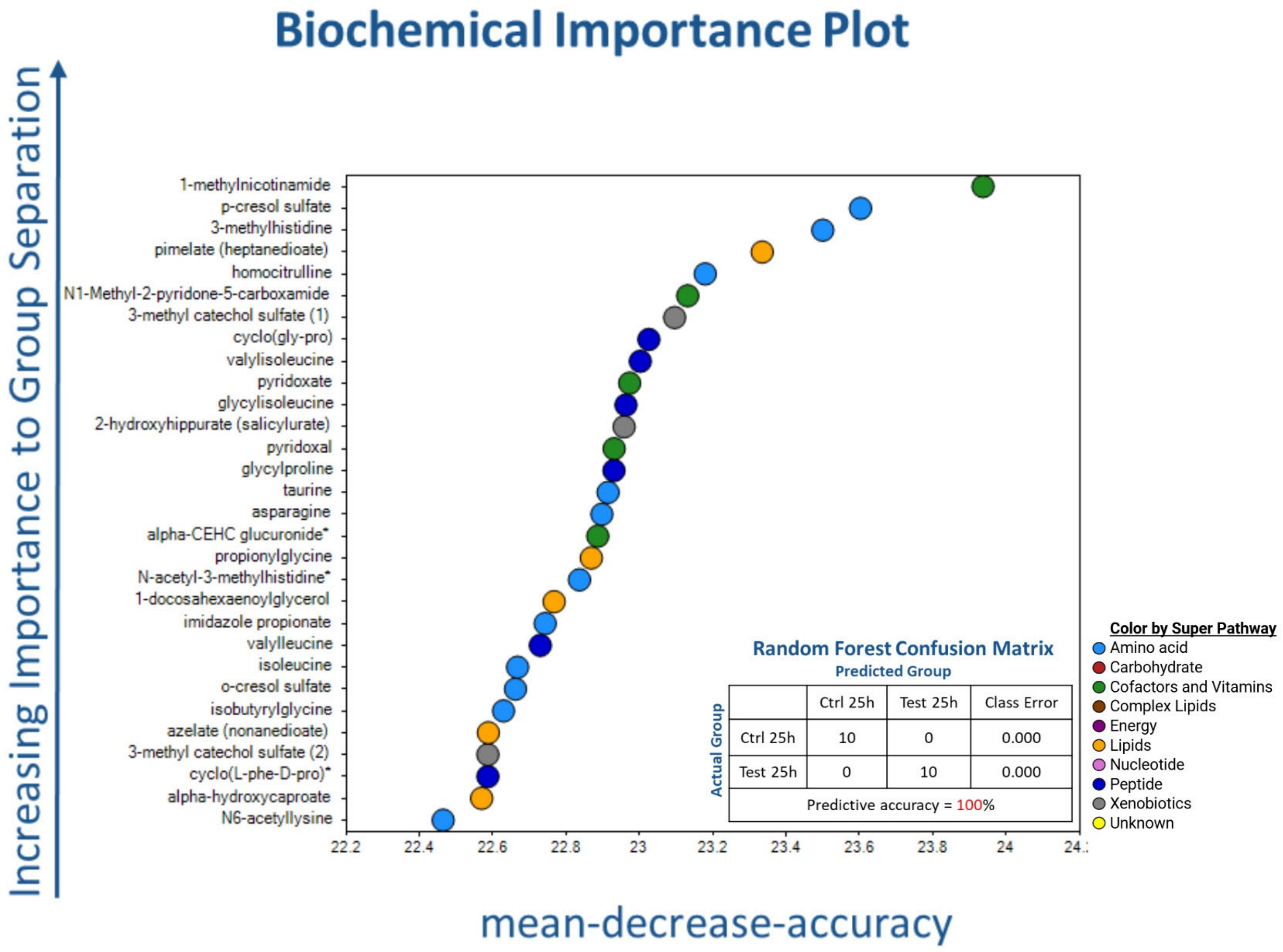

Supplement: Supplementary file 1 [file metabolites-16-00397-s001.zip › metabolites-4303417-supplementary.pdf]
